# Anatomical and volumetric description of the guiana dolphin (*Sotalia guianensis*) brain from an ultra-high-field magnetic resonance imaging

**DOI:** 10.1007/s00429-024-02789-1

**Published:** 2024-04-25

**Authors:** Kamilla Avelino-de-Souza, Heitor Mynssen, Khallil Chaim, Ashley N. Parks, Joana M. P. Ikeda, Haydée Andrade Cunha, Bruno Mota, Nina Patzke

**Affiliations:** 1 Instituto de Ciências Biomédicas, Universidade Federal do Rio de Janeiro, Rio de Janeiro, 21941-590 Brazil; 2 Laboratório de Biologia Teórica e Matemática Experimental (MetaBIO), Instituto de Física, Universidade Federal do Rio de Janeiro, Rio de Janeiro, 21941-909 Brazil; 3 Rede Brasileira de Neurobiodiversidade, Instituto de Física, Universidade Federal do Rio de Janeiro, Rio de Janeiro, 21941-909 Brazil; 4 LIM44, Faculdade de Medicina, Hospital das Clínicas HCFMUSP, Universidade de São Paulo, São Paulo, SP Brazil; 5 Renaissance School of Medicine, Stony Brook University, New York, USA; 6 Laboratório de Mamíferos Aquáticos e Bioindicadores Professora Izabel M.G do N. Gurgel (MAQUA), Faculdade de Oceanografia, Universidade do Estado do Rio de Janeiro, Rio de Janeiro, Brazil; 7 Departamento de Genética, Instituto de Biologia Roberto Alcântara Gomes, Universidade do Estado do Rio de Janeiro, Rio de Janeiro, Brazil; 8 Faculty of Medicine, Institute of Mind, Brain and Behavior, Health and Medical University, Olympischer Weg 1, 14471 Potsdam, Germany

**Keywords:** Brain evolution, Dolphin brain, MRI, Comparative neuroanatomy

## Abstract

The Guiana dolphin (*Sotalia guianensis*) is a common species along Central and South American coastal waters. Although much effort has been made to understand its behavioral ecology and evolution, very little is known about its brain. The use of ultra-high field MRI in anatomical descriptions of cetacean brains is a very promising approach that is still uncommon. In this study, we present for the first time a full anatomical description of the Guiana dolphin’s brain based on high-resolution ultra-high-field magnetic resonance imaging, providing an exceptional level of brain anatomical details, and enriching our understanding of the species. Brain structures were labeled and volumetric measurements were delineated for many distinguishable structures, including the gray matter and white matter of the cerebral cortex, amygdala, hippocampus, superior and inferior colliculi, thalamus, corpus callosum, ventricles, brainstem and cerebellum. Additionally, we provide the surface anatomy of the Guiana dolphin brain, including the labeling of main sulci and gyri as well as the calculation of its gyrification index. These neuroanatomical data, absent from the literature to date, will help disentangle the history behind cetacean brain evolution and consequently, mammalian evolution, representing a significant new source for future comparative studies.

**Supplementary Information:**

The online version contains supplementary material available at 10.1007/s00429-024-02789-1.

## Introduction

The Guiana dolphin (*S. guianensis*) is a small cetacean present from the warm waters of Nicaragua in Central America to the colder waters of Southern Brazil in South America. The species is found in shallow estuarine and coastal waters (Flores et al. 2018).

The Guiana dolphin is part of the genus *Sotalia* (family Delphinidae), which is composed of two species: marine *Sotalia guianensis* and riverine *Sotalia fluviatilis*. These very similar species were recently separated based on morphometric differences in skull shape (Monteiro-Filho et al. 2002) and subsequently, by molecular analyses using mitochondrial DNA sequences (Cunha et al. 2005).

Numerous studies characterize the behavior of this species, including cooperative fishing, signature whistles, parental behavior, and even interspecies interactions with dogs (Pierry et al. 2023; Tardin et al. 2013; Lima and Le Pendu 2014; Tardin et al. 2013; Flores et al. 2018). Additionally, morphological investigations have examined the relationship between body size and skull size (Drago et al. 2021; Ramos et al. 2010) as well as with other anatomical structures such as eyes, reproductive organs, and electroreception structures (Becegato et al. 2015; Czech-Damal et al. 2012; da Silva and Best 1996; Rodrigues et al. 2022). Yet, while the Guiana dolphin’s behavior and anatomy have been the subject of several previous investigations, research on its brain morphology, to the best of our knowledge, is not available.

In a broader context, in recent years, there has been a growing interest in cetacean neuroanatomy, due in particular to their enlarged brain size and highly convoluted neocortex (Marino 2002; 2004a, b, c; Manger et al. 2012; Cozzi et al. 2017). This has motivated many research groups to adopt more modern and practical anatomical techniques, leading to the first studies on cetacean brains using magnetic resonance imaging (MRI) (e.g.Marino et al. 2001a, 2002, 2004a, c; Montie et al. 2007, 2008; Oelschläger et al. 2008). Despite recent advancements in the study of cetacean brains, there is still ample opportunity for improvement, particularly given the vast diversity of species within the Cetacea group. It is crucial to study more species to develop a more comprehensive understanding of brain evolution within this group.

In addition to its usefulness in examining brain structures and revealing functional and evolutionary patterns, MRI is also commonly used to identify brain tissue pathologies in humans and other species (Tofts 2005; Johnson et al. 2012; Ibarretxe-Bilbao et al. 2011; Hitoshi et al. 1991). This is particularly relevant for cetaceans, as it would be important to identify and characterize pathologies that are known to affect the central nervous system and cause abnormal behaviors, such as neurobrucellosis (Sánchez‐Sarmiento et al. 2019; Hernández-Mora et al. 2008), taxoplasmosis (Guardo et al. 2010; Roe et al. 2013), and those caused by the morbilliviruses (Giorda et al. 2022; Díaz-Delgado et al. 2019). The latter, in particular, have been linked to epidemics and mass mortality events in the Guiana dolphin (Cunha et al. 2021; Flach et al. 2019; Groch et al. 2018, 2020). However, accessing the brain to examine the presence of lesions histologically and/or genetically is not always feasible. In this regard, MRI studies could provide an additional means of investigating disorders, of postmortem carcasses or extracted whole brains, but also in smaller cetaceans living under human care (Ridgway et al. 2006). Such studies can provide valuable insights and may contribute to the development of more effective management and conservation strategies. Nonetheless, a thorough understanding of the normal healthy brain structure is necessary to investigate the impacts of external threats on cetacean brains.

This study aims to expand current knowledge of cetacean neuroanatomy by providing the first imaging analysis of the Guiana dolphin brain. Here, we present an MRI-based detailed description of the Guiana dolphin brain with labeled images in the coronal, sagittal, and horizontal planes using a 7T ultra-high field MRI scanner. The use of this methodology allows for a more detailed identification and analysis of diverse structures. The resulting comprehensive dataset serves as a valuable resource for future comparative studies that can enhance our understanding of cetacean neuroanatomy and brain evolution.

## Materials and methods

### Specimen

**Fig. 1:**
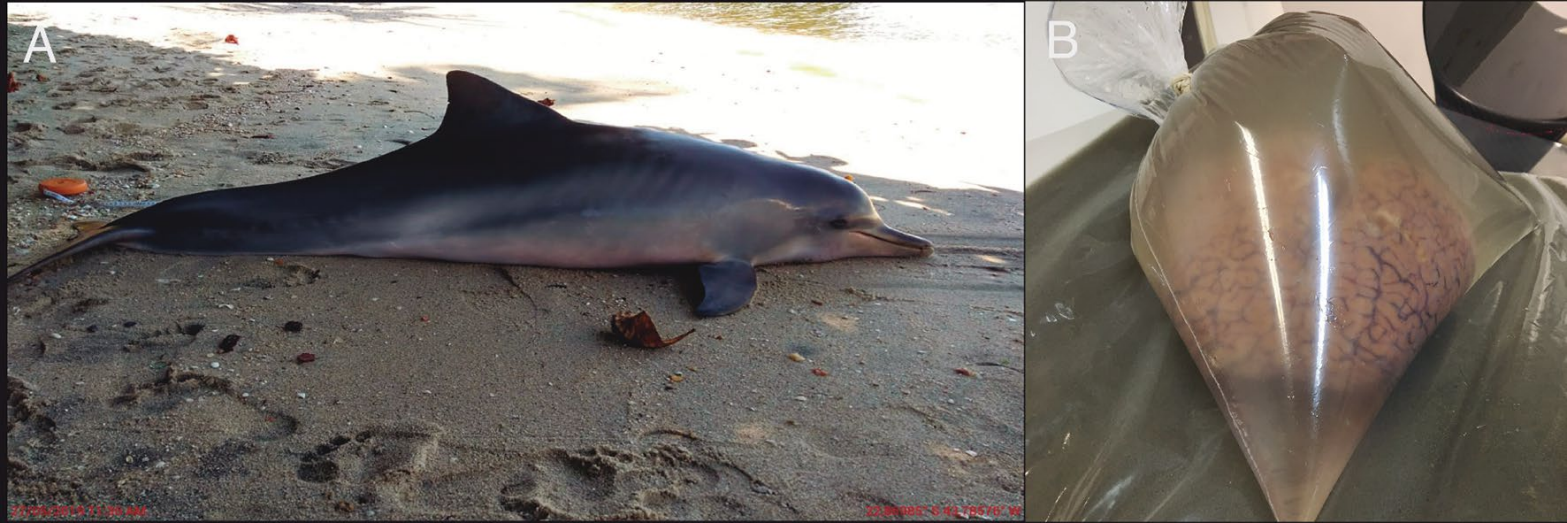
**a** Stranded adult female Guiana dolphin from Baía de Sepetiba, Rio de Janeiro, Brazil. The body length is 174 cm, **b** Guiana dolphin brain in a plastic bag filled with sodium azide phosphate buffer prior to MRI scanning

The brain of an adult female Guiana dolphin (*Sotalia guianensis*), 174 cm long, was acquired opportunistically following a post-mortem investigation of the animal, which stranded at Baía de Sepetiba in Rio de Janeiro, Brazil (Fig. 1a). The specimen was collected with authorization from the Chico Mendes Institute for Biodiversity Conservation (ICMBio) and the Brazilian Institute of Environment and Renewable Natural Resources (IBAMA). Specifically, the collection was conducted under the Biodiversity Information and Authorization System (SISBIO) permit No 50104 and the authorization for collection and transport of biological materials (ABIO) permit No 755/2016 respectively. This study was approved by the Committee on Ethical Animal Use of the Science Center of the Federal University of Rio de Janeiro (process number 01200.001568/2013-87).

At the time of the dissection, the carcass was in fresh condition. The brain was removed from the skull, and it was subsequently immersion-fixed in 4% paraformaldehyde in 0.1 M phosphate buffer (PB) for 5 days at 4 °C to allow proper fixation (Dell et al. 2016). It was then stored in 0.1 M PB with 0.1% sodium azide at 4 °C to preserve the tissue until further use.

Before scanning, the brain was removed from the fridge and left overnight in the scanner room to allow the tissue to adjust to the ambient temperature. Subsequently, the ex-situ brain was placed in a plastic bag that was filled with 0.1 M PB with 0.1% sodium azide to ensure a better fit with the scanner head coil (Fig. 1b).

## MRI protocols

The MR images were acquired in a 7 Tesla Scanner (Classic Magneton, Siemens Healthcare, Erlangen, USA) equipped with a 32-channel head coil with single radiofrequency transmission (Nova Medical, Wilmington, USA) at the “Image Platform in the Autopsy Room” (PISA) facilities of the School of Medicine at the University of São Paulo.

To enhance the recognition and differentiation of brain structures, high-resolution coronal anatomical images were acquired with two protocols: bi-dimensional turbo spin echo sequence (2D-TSE) and a single slab three-dimensional turbo spin echo sequence (3D-SPACE). The 2D-TSE acquisition parameters were: TR/TE = 6000/48 ms; in-plane spatial resolution = 0.27 mm (FoV = 195 × 146; matrix 720 × 540); 55 slices with slice thickness = 2 mm (no GAP); Echo Train Length (ETL) = 5; BW = 224 Hz/px; number of signal averages (NSA) = 3; variable excitation pulse starts with 140˚ and no parallel imaging factor. The 3D-SPACE acquisition parameters were: TR/TE = 2000/109 ms and fast recovery; 0.38 mm isotropic spatial resolution (FoV = 192 × 144 mm; matrix 512 × 384); 320 slices in slab with slice thickness = 0.38 mm; variable excitation pulse starts with 120˚; ETL = 40; BW = 610 Hz/px and parallel imaging factor (GRAPPA) = 2. The acquisition time was 33 min for 2D-TSE and 1 h and 45 min for 3D-SPACE. In this study, we used turbo spin echo acquisitions to mitigate geometric distortion and susceptibility artifacts due to air bubbles, which is critical to our analysis.

## Quantitative analysis

### MRI analysis

Anatomical plane adjustments and morphological/volumetric data acquisition were performed using the software OsiriX MD v.12.0.3 (Pixmeo, Geneva, Switzerland; Rosset et al. 2004). Brain structures were delineated through manual segmentation of the brain slides using a touch display and digital pen (Wacom Mamboo, Kazo, Japan). The OsiriX pencil tool was employed based on cetacean neuroanatomy literature and our knowledge to create the regions of interest (e.g.Huggenberger et al. 2019; Cozzi et al. 2017; Marino et al. 2001a, b; 2002; 2004a, b, c;Oelschläger et al. 2008, 2010; Morgane and Jacobs 1972, Morgane et al. 1980. In addition, we utilized a grayscale inversion filter from OsiriX to make the fixed tissue T2-weighted images appear similar to T1-weighted images (white matter brighter than gray matter) and ensure more accurate manual tracing of the images. The volumes of the following structures were estimated: cortical gray matter (GM), cerebral white matter (WM), amygdala, hippocampus, ventricles, brainstem, superior and inferior colliculi, thalamus, and cerebellum.

**Fig. 2:**
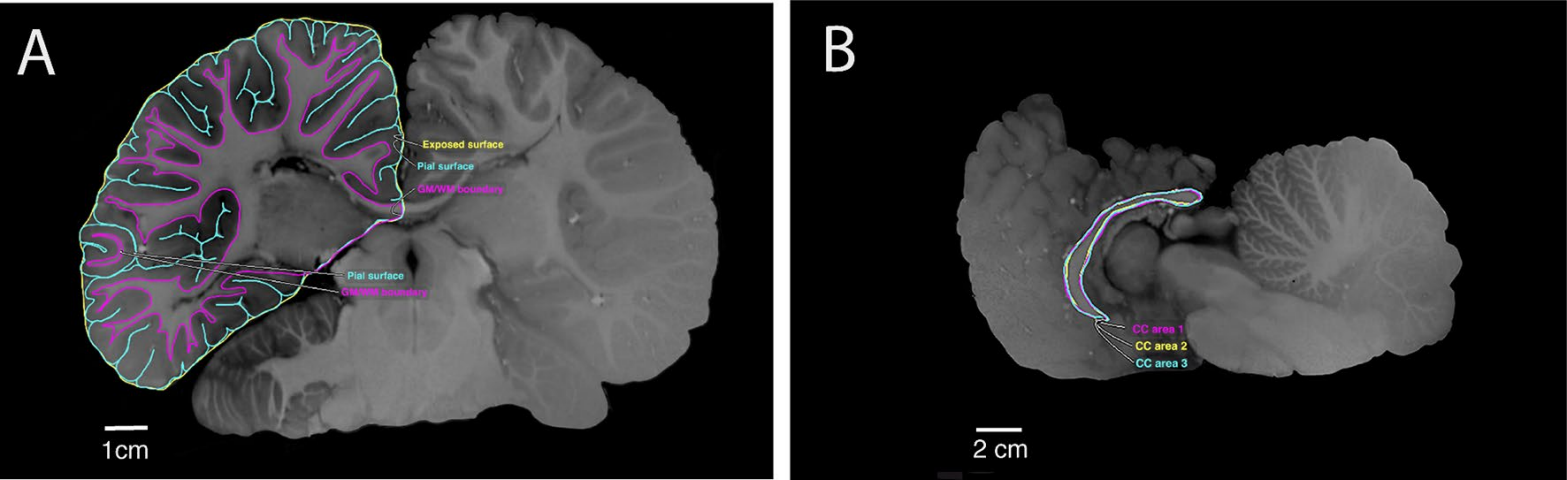
**A **Manual tracings for exposed (yellow), pial (blue), and GM/WM interface (pink) perimeters. Note that the pial surface areas include cortical sulci and gyri whereas the external contours do not. Scale bar = 1 cm, **B** Manual tracing of the callosal boundaries on the most midsagittal section of the Guiana dolphin brain. The cc area was the average of three measurements for the section (blue, pink, and yellow). Scale bar = 2 cm

In order to calculate cortical area volumes, we delineated gray and white matter in every 5th slice, resulting in a distance of 1.9 mm between each adjacent slice and a total of 49 contoured brain slices. Subcortical structures, such as the thalamus, were included within the contour to avoid crossing the white matter at an arbitrary location (Fig. 2a). This inclusion procedure is similar to what is performed in automatic segmentation for humans (Nitzberg and Shiota 1992; Bullmore et al. 1995; Dale et al. 1999; Zhang et al. 2001). Previous studies (Manger et al. 2012; Kazu et al. 2014) used segmentation with open lines, which implicitly creates a straight connection between the initial and final points for the calculation of the contour interior area. This area is essential for the correct calculation of the geometrical values of pial and exposed surfaces.

As a result, we obtained three detailed cortical surfaces: (1) the exposed, (2) pial and (3) the GM/WM boundary (Fig. 2a). From these surfaces, we calculated exposed cortical and pial areas, GM and WM volumes, and total volume, using the geometric approximation previously described in Ribeiro et al. (2013). This approximation accounts for the varying slope of the lateral surfaces between contours as one moves along the stack of slices and will always converge to the correct value for sufficiently small slice thickness. In contrast, previous estimates of areas and volumes assumed strictly vertical lateral surfaces, inducing substantial systematic errors in area estimates that persist even for arbitrarily thin slices (Avelino-de-Souza 2023, Appendix D; See Supplementary material for details).

The average cortical thickness was calculated as the difference between the total and white matter volumes divided by the total area (Mota and Herculano-Houzel 2015).

To calculate the volume of the brainstem and subcortical structures, such as the hippocampus, amygdala, and thalamus, we measured the perimeters of every 5th slice in the 3D acquisition. This resulted in a final slice thickness of 1.9 mm. To calculate the volume for the superior and inferior colliculi, cerebellum, and ventricular system, we delineated the perimeters of these structures in every slice of the 2D coronal acquisition, corresponding to a slice thickness of 2.0 mm and 35 contoured slices. The volumes and areas for all the structures were calculated using the formula described by Ribeiro et al. (2013).

The MRI scans were imported into Adobe Illustrator (version 27.7, Adobe Inc.) for final adjustments, grouping, and labeling of all structures. MRI images without labeling, along with representative slices of scans with no contrast adjustments, are available as Supplementary material.

## Gyrification index, corpus callosum area/brain mass ratio, and encephalization quotient

The Encephalization Quotient parameter (EQ) assumes that brain mass increases with body mass in a manner that can be described by a power law (Jerison 1973). EQ enables the calculation of the expected brain mass for a given body mass, as well as the variation between the expected and observed brain masses. Here, we calculated EQ using the general mammalian regression equation described in Manger (2006).

Additionally, the Gyrification Index (GI)—a measure of cortical folding—was calculated using the methodology described in Zilles et al. (1989). Briefly, GI is calculated by dividing the pial surface area by the external surface area. The pial surface area includes cortical sulci and gyri whereas the external surface area does not (Fig. 2a). Because both contours include the subcortical regions, the GI should suffer a reduction due to the smoothness of that region. These surface areas were calculated by the method described above (Ribeiro et al. 2013).

The corpus callosum area (CCA) was determined by the manual tracing of the callosal boundaries of the midsagittal section (Fig. 2b) of the 3D acquisition. The final value was the average of three measurements of the section. The corpus callosum area/brain mass ratio (CCA: BM) was calculated using two methodologies: Tarpley and Ridgway’s equation applies the simple division of the CCA (mm^2^) by the brain mass (g) whereas Manger’s equation divides the square root of CCA (mm^2^) by the cubic root of brain mass (g) (Tarpley and Ridgway 1994; Manger et al. 2010).

## Results

### Anatomical and volumetric analysis

#### Whole brain

The Guiana dolphin weighs 45.5 kg, with a brain mass of 716.4 g, including meninges. The brain volume extracted from MRI scans was 659.052 cm^3^, which was converted to 682.78 g (brain volume (cm^3^) x brain tissue-specific gravity (1.036 g/cm^3^; Stephan et al. 1981). The difference observed between the calculated brain volume from manual segmentations and the fresh brain weight may be attributed to the meningeal weight.

Based on the measurements described in the methods, the EQ calculated for the Guiana dolphin was 4.69. This number falls well within the prediction interval described for other delphinids – from 2.01 to 6.32 and closely aligns with the EQ values for the bottlenose dolphin (*Tursiops truncatus*; 4.47) and the Atlantic white-sided dolphin (*Lagenorhynchus acutus*; 4.43) (Manger 2006).

## External anatomical features

The general morphology of the Guiana dolphin brain is similar to other species previously studied within the Delphinidae family, including the common dolphin (*Delphinus delphis*) (Marino et al. 2001b, 2002; Oelschläger et al. 2010) and the bottlenose dolphin (*Tursiops truncatus*) (Marino et al. 2001a, 2004a).

**Fig. 3:**
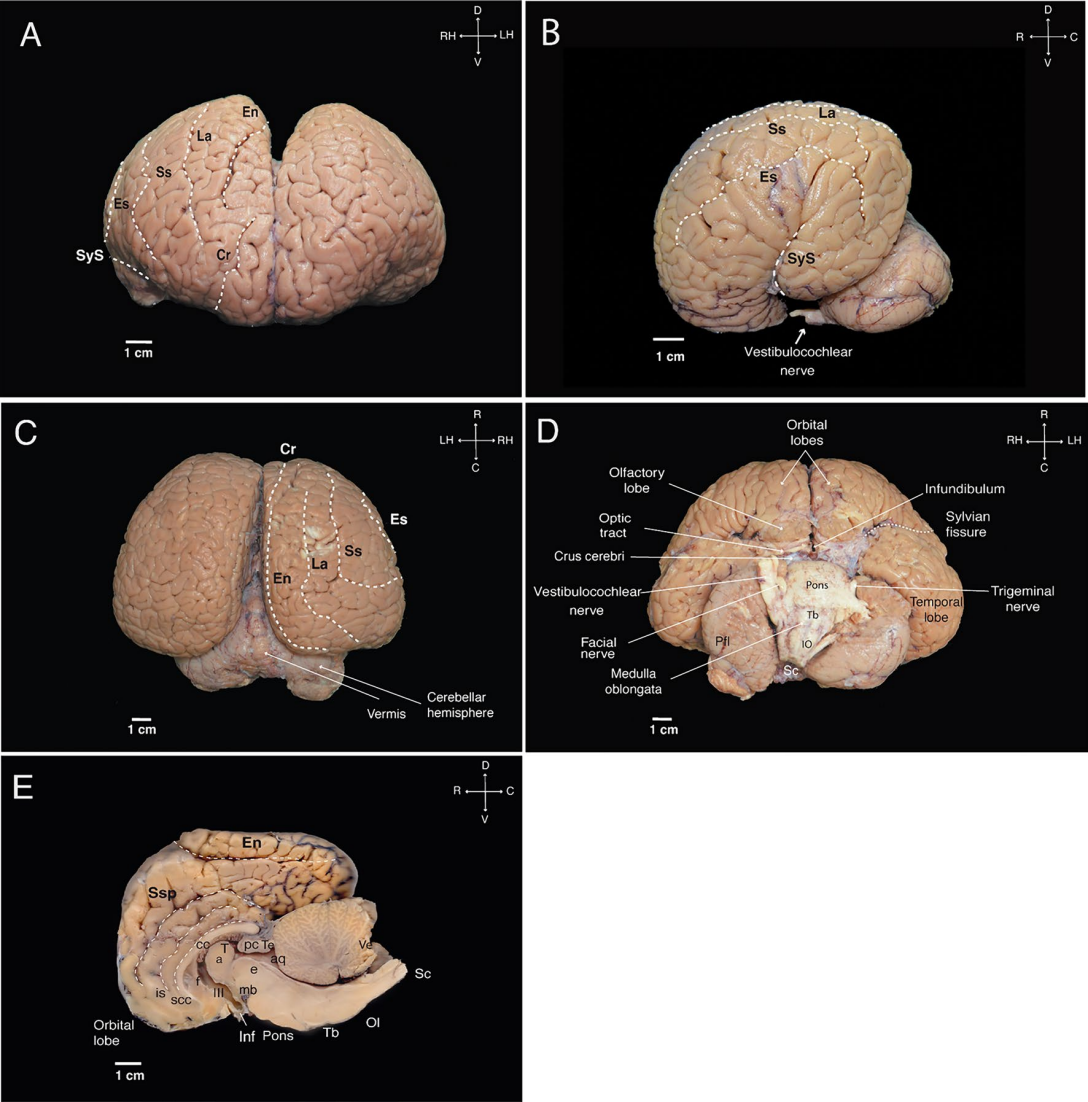
External aspects of the Guiana dolphin fresh brain in frontal (**A**), lateral (**B**), dorsal (**C**), ventral (**D**), and mid-sagittal (**E**) views. Scale bar = 1 cm. Anatomical directions: **D** (dorsal), V (ventral), R (rostral), and C (caudal). For other abbreviations see list

The Guiana dolphin brain exhibits the characteristic cetacean brain shape. It is large and globular, with a highly convoluted telencephalon that is wider than it is long (width: 15 cm; length: 12 cm, height: 6.8 cm). The forebrain is rostroventrally rotated, resembling the shape of a boxing glove (Fig. 3 a–e) (Spocter et al. 2017; Morgane 1980). As is typical of odontocetes, the olfactory bulb and tract are absent (Oelschläger 2008; Oelschläger et al. 2010; Marino et al. 2001a, b).

The most rostroventral aspect of the Guiana dolphin brain is defined by the juxtaposed orbital lobes (Fig. 3a). Towards the ventrocaudal portion of the orbital lobes, the olfactory lobes become apparent. Continuing caudally, only the most ventral region of the diencephalon is visible, delineated by the optical tracts and a residual part of the infundibulum. The optic chiasma and the infundibulum were damaged during the dissection of the brain, exposing the opening of the third ventricle. An outward bulging of the corpora mammillaria is missing. The rest of the diencephalon is enveloped by massive telencephalic hemispheres (Fig. 3d).

At the brainstem level, we could observe the most ventral part of the mesencephalon, represented by the crus cerebri, as well as the pons bulging just inferior to it as a wide and well-developed structure. The trigeminal nerve emerges laterally from the pons, while the facial and vestibulocochlear nerves arise more caudally at the transition to the medulla oblongata (Fig. 3d). As is typical of dolphins, the vestibulocochlear nerve exhibits the highest thickness compared to the other cranial nerves. Moving to the caudal aspect of the pons, the trapezoid body becomes apparent. The medulla oblongata, positioned immediately below the trapezoid body, is clearly distinguishable due to the presence of the inferior olives protruding on its ventral surface. The structure then narrows and transitions into the spinal cord (Fig. 3d). From the dorsal view, the brainstem is covered by the telencephalon and the cerebellum (Fig. 3c). Although voluminous, the cerebellum is not as wide as the cerebral cortex. It consists of a narrow vermis and two adjacent large hemispheres, which present a flattened appearance as they extend under the voluminous cerebral hemispheres (Fig. 3b–e). The very large paraflocculus was evident in the posterior lobe of the cerebellum (Fig. 3d).

Various subcortical structures were identified from the midsagittal view and these internal components will be further described in the subsequent sections.

## Telencephalon

The telencephalic volume was 518.40 cm^3^ and accounted for 78.40% of the overall brain volume. The right hemisphere's total volume, comprising both white matter and gray matter, was 265.089 cm^3^, slightly surpassing the left hemisphere, which had a total volume of 253.317 cm^3^. The underlying total white matter volume amounted to 198.93 cm^3^, with 106.76 cm^3^ assigned to the right hemisphere and 92.18 cm^3^ to the left hemisphere. For the gray matter, the total volume was 319.47 cm^3^, with the right hemisphere exhibiting a marginally greater volume of 160.13 cm^3^ compared to the left hemisphere’s 158.33 cm^3^ (See Table 1). The average cortical thickness calculated was 2.26 mm, which is close to, but slightly higher than the reported values of 1.99 and 2.02 mm obtained for other cetacean species: the Risso’s dolphin (*Grampus griseus*) and the pilot whale *(Globicephala macrorynchus*) respectively (Hofman 1985). Additionally, our measurement also slightly exceeds values reported by Furutani (2008) for thickness across different cortical areas, ranging from 1.7 to 2.0 mm in the Risso’s dolphin, 1.5–1.8 mm in the striped dolphin (*Stenella coeruleoalba*) and 1.6–1.8 mm in the bottlenose dolphin.

**Table 1:** Measurements of regions of interest (ROIs) for the *S. guianensis* brain

ROI	Measurements	Result
Whole brain and external anatomy	Maximal height	6.8 cm
	Maximal width	15 cm
	Antero-posterior length	12 cm
	Total volume	659.05 cm^3^
	Mass	682.78 g*
Telencephalon	Gray matter (GM) volume	319.47 cm^3^
	White matter (WM) volume	198.93 cm^3^
	Hippocampus volume	0.66 cm^3^
	Fraction of total brain volume	0.10%
	Left-side volume	0.329 cm^3^
	Right-side volume	0.331 cm^3^
	Amygdala volume	0.95 cm^3^
	Fraction of total brain volume	0.14%
	Left-side volume	0.49 cm^3^
	Right-side volume	0.46 cm^3^
Diencephalon	Thalamus volume	15.04 cm^3^
	Fraction of total brain volume	2.28%
	Left-side volume	7.27 cm^3^
	Right-side volume	7.76 cm^3^
Cerebellum	Volume	113.82 cm^3^
	Fraction of total brain volume	17.27%
Corpus callosum	Cross-sectional area	1.132 cm^2^
Ventricular system	Volume	6.263 cm^3^
	Fraction of total brain volume	0.95%
Brainstem	Volume	25.02 cm^3^
	Fraction of total brain volume	3.80%
	Superior colliculi volume	0.512 cm^3^
	Fraction of total brain volume	0.078%
	Left-side volume	0.276 cm^3^
	Right-side volume	0.263 cm^3^
	Inferior colliculus volume	2.68 cm^3^
	Fraction of total brain volume	0.40%
	Left-side volume	1.352 cm^3^
	Right-side volume	1.211 cm^3^

*The conversion from brain volume to weight units was made by multiplying the total brain volume by the brain tissue-specific gravity (1.036 g/cm^3^) (Stephan et al. 1981)

The GI calculated for the Guiana dolphin was 3.08, which is relatively high compared to other mammals, including primates, afrotherians, carnivores, and artiodactyls (Fig. 7b). However, it is lower than the average GI of 5.43 reported for cetaceans in previous studies (Manger et al. 2012). Moreover, a strong correlation seems to exist between GI and brain mass in primates, carnivores, and artiodactyls (exponent of 0.1531 ± 0.006; r^2^ = 0.88; p < 0.0001, based on data from Zilles et al. 1989; Pillay and Manger 2007; Manger et al 2012; Kazu et al. 2014). Conversely, no such correlation has been found in cetaceans (Manger et al. 2012), including the Guiana dolphin analyzed here (exponent of 0.119 ± 0.095; r^2^ = 0.23; p = 0.287). Therefore, it seems that in cetaceans, gyrencephaly does not increase with brain mass.

The organization of gyri and sulci was similar to that previously described for other cetacean species. On the lateral surface (Fig. 3b), we delineated the Sylvian fissure (lateral sulcus), which exhibits an unusual orientation in cetaceans, including the specimen here described. It is positioned almost perpendicular to the rostro-caudal axis of the brain, at a nearly right angle with respect to the animal’s body axis. The Sylvian fissure was also delineated in the rostral and ventral views (Fig. 3a, d).

Surrounding the Sylvian fissure were the major lateral sulci: the ectosylvian (es), suprasylvian (ss), and lateral (La) sulci (synonymous with inferior, intermediate, and superior lateral sulcus, respectively). From the dorsal and frontal views, we could also readily identify the entolateral sulcus (en; syn. paralimbic sulcus) and the cruciate sulcus (cr) (Fig. 3a, c). At the medial surface of the hemispheres, we observed the entolateral sulcus, suprasplenial sulcus (Ssp; syn. limbic cleft), the intercalate sulcus (is), and the sulcus of corpus callosum(scc) (Fig. 3e).

**Fig. 4:**
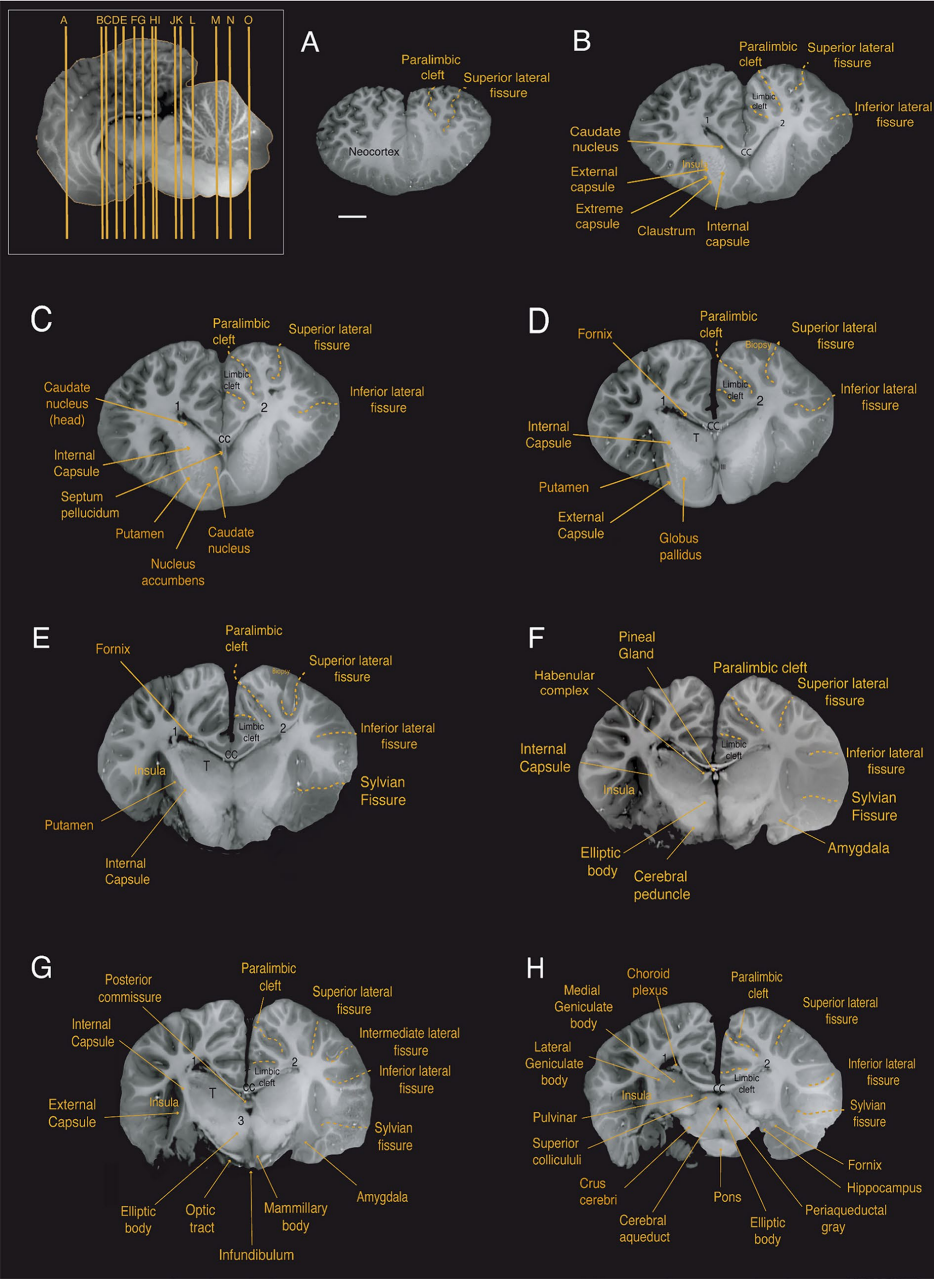

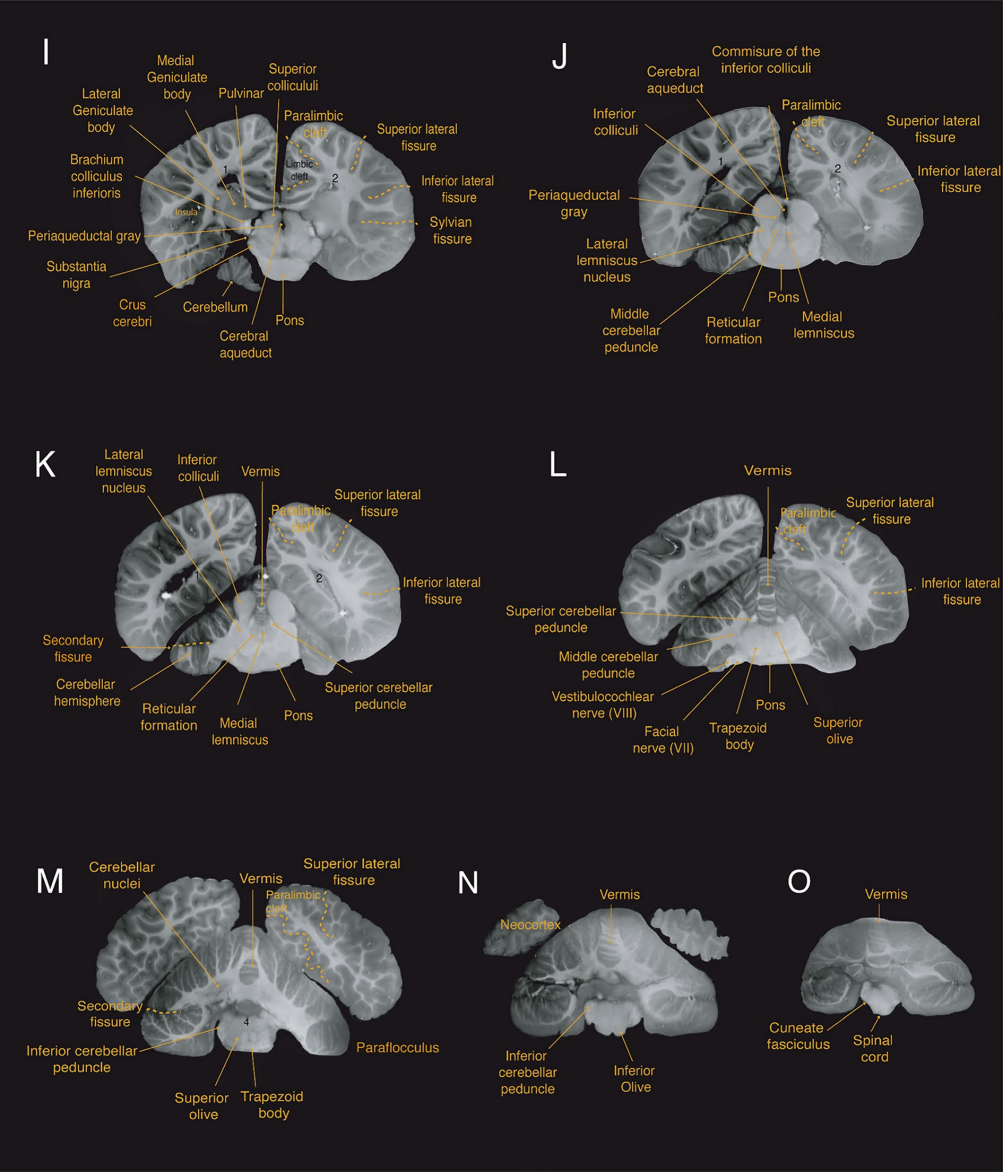
Coronal magnetic resonance imaging (MRI) scans of the Guiana dolphin brain at 1.9mm interval—from anterior to posterior axis; T2 weighted; MRI grayscale inverted. Scale bar = 2 cm. Top left: Illustration of the position of the brain in the sagittal plane. For abbreviations see list

**Fig. 5:**
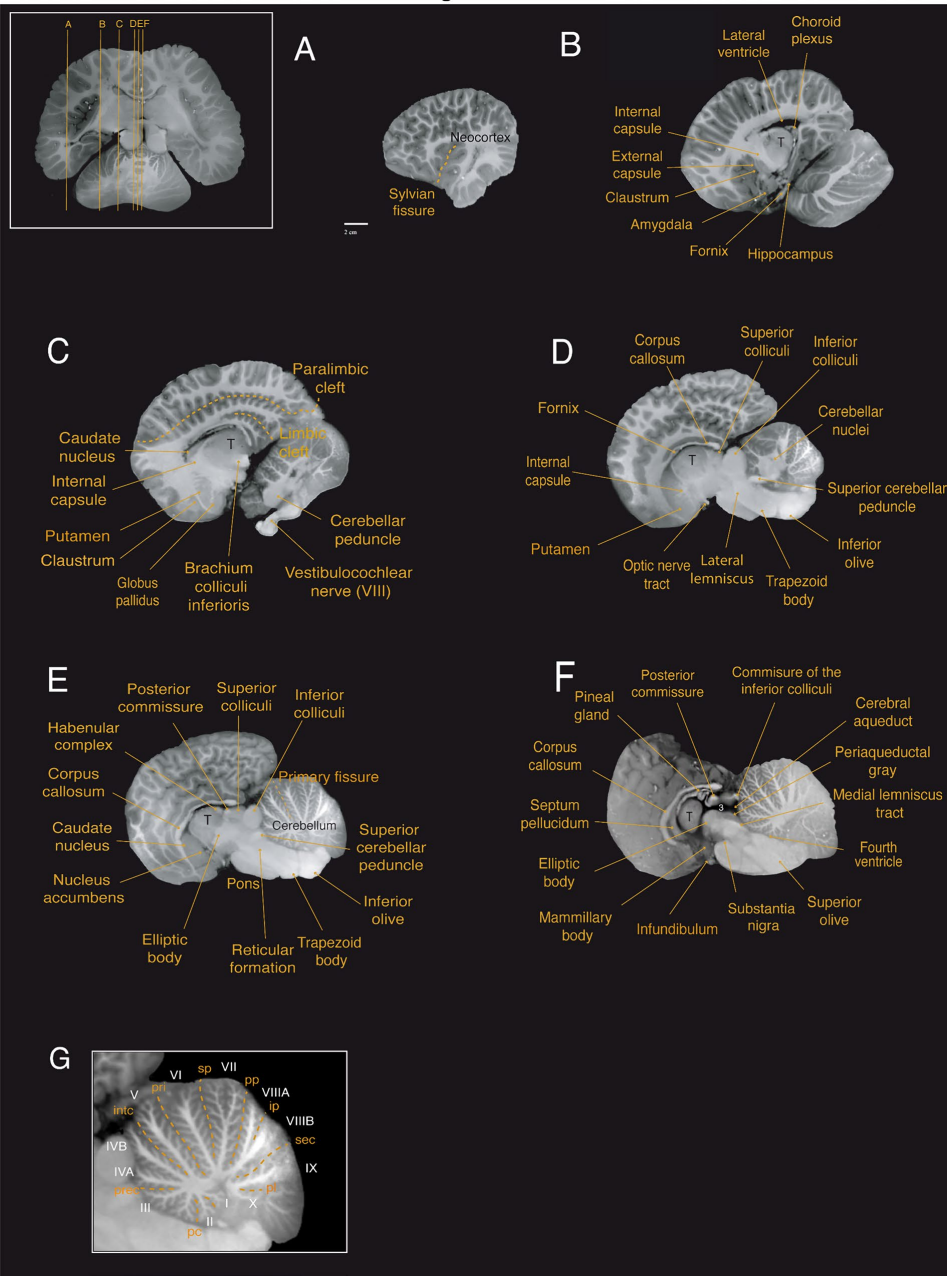
Sagittal magnetic resonance imaging (MRI) scans of the Guiana dolphin brain at 1.9 mm interval—from to axis; T2 weighted; MRI grayscale inverted. Scale bar = 2 cm. Top left: Illustration of the position of the brain in the horizontal plane. In the bottom, the midsagittal cerebellar section demonstrates the lobules and arborization patterns of the vermis of the structure. Cerebellar lobules are labeled in white, and sulci are labeled in yellow. For abbreviations see list

**Fig. 6:**
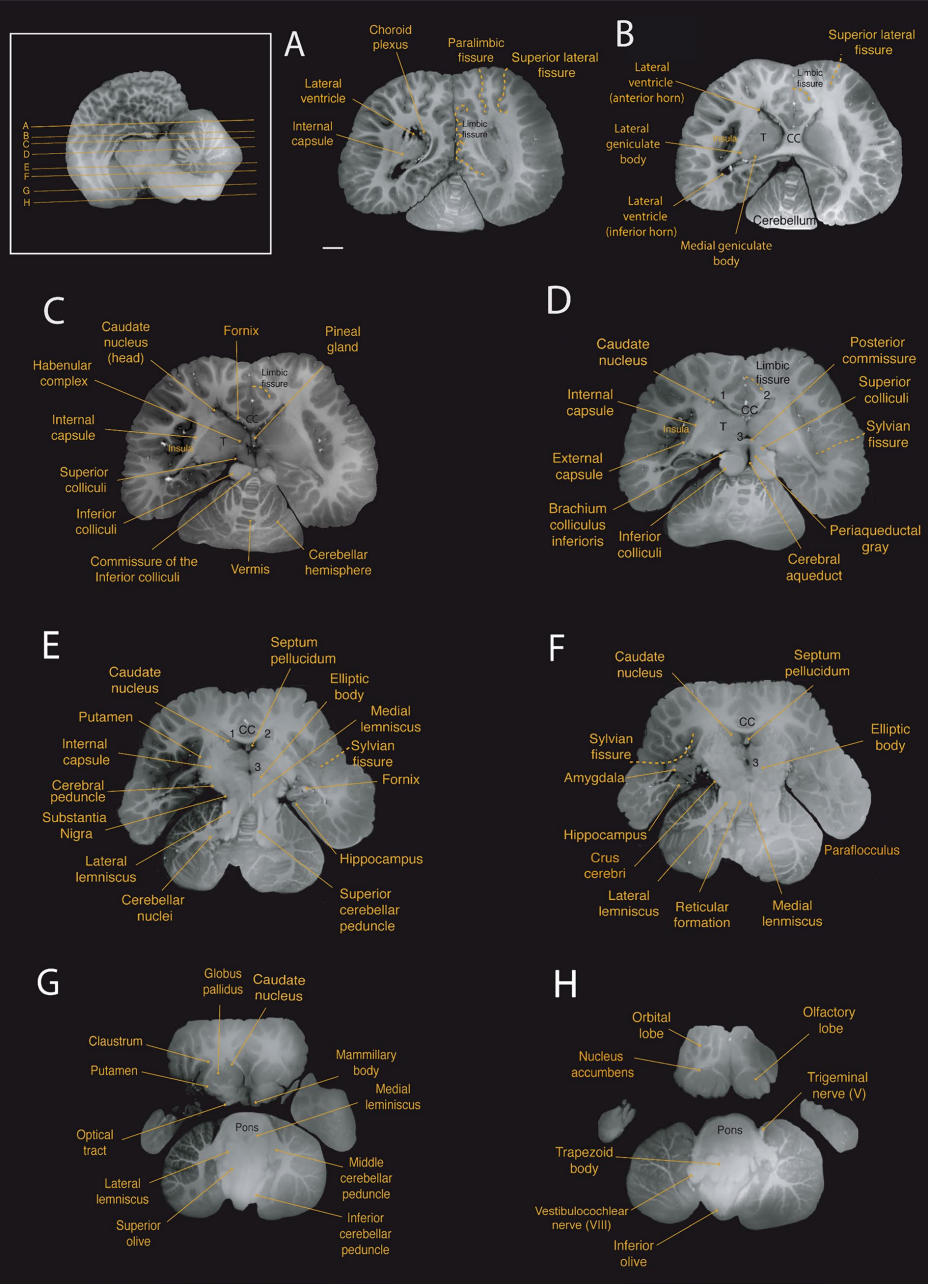
Horizontal magnetic resonance imaging (MRI) scans of the Guiana dolphin brain at 1.9 mm interval—from to axis; T2 weighted; MRI grayscale inverted Scale bar = 2 cm. Top left: Illustration of the position of the brain in the sagittal plane. For abbreviations see list

At the subcortical level, we observed the corpus striatum, formed by the caudate nucleus and putamen, located lateral to the thalamus. The striatum is partially divided by a well-developed internal capsule (Figs. 4b–e; 5c–e; 6c–g). At the ventral portion of the striatum, the caudate nucleus and the putamen remain connected allowing for the identification of what is presumably the nucleus accumbens; however, this identification requires histological verification (Figs. 4c; 5e; 6h). The globus pallidus, located medial to the posterior portion of the putamen and separated from it by a thin sheet of white matter, has a slightly paler appearance compared to surrounding structures, which can be attributed to a higher concentration of myelinated fibers (Figs. 4d; 5c; 6g).

The claustrum was identified as a thin and elongated sheet of gray matter situated medial to the anterior insular cortex and lateral to the striatum, as previously reported for the bottlenose dolphin (Cozzi et al. 2014). “Cell islands”, as described in the bottlenose dolphin (Baizer et al. 2014), could not be identified in our MRI scans. The claustrum was separated laterally by a very thin extreme capsule from the insular cortex and medially by a stronger developed external capsule from the putamen (Figs. 4b;5b,c; 6g).

**Fig. 7:**
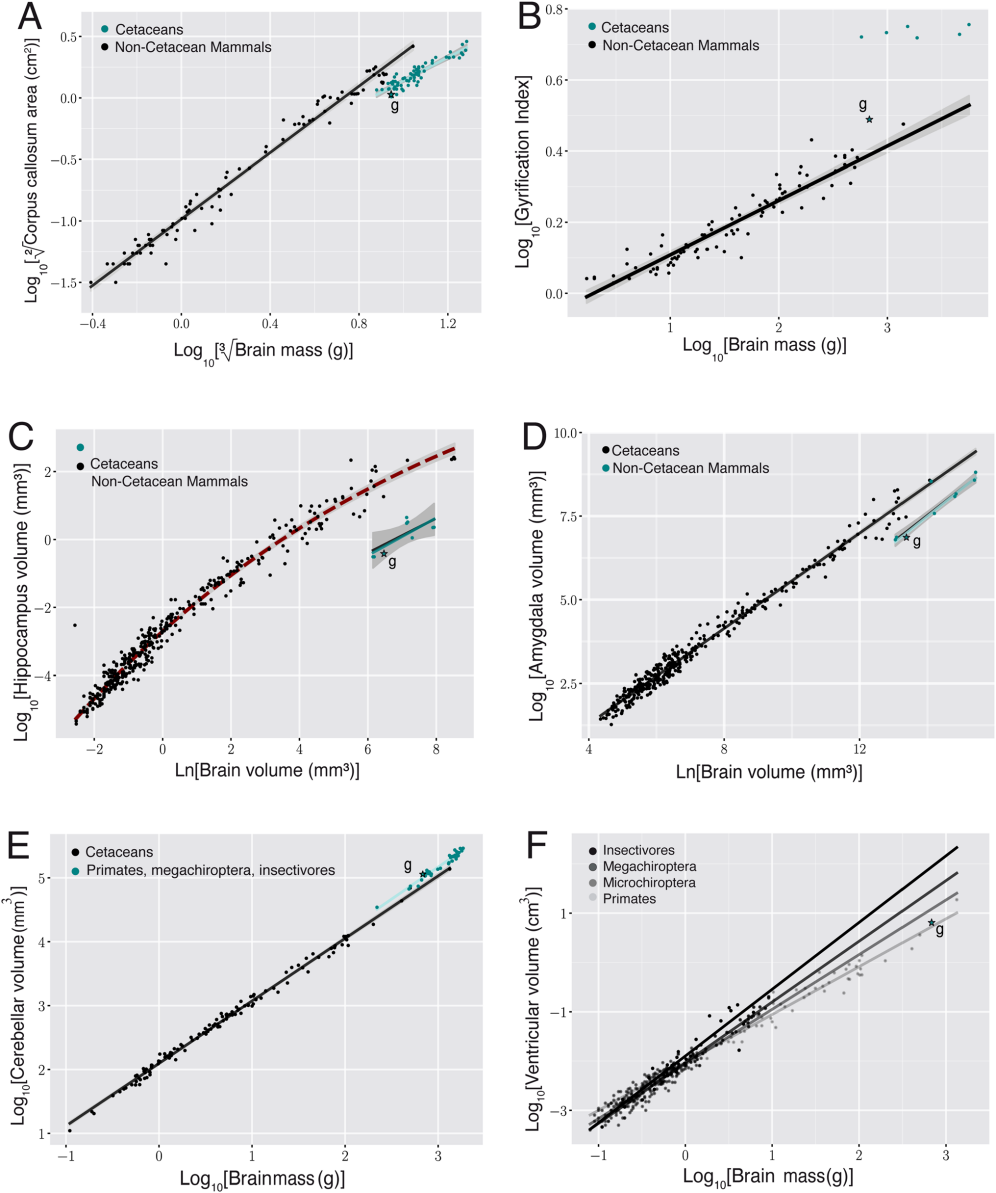
**a** When regressing the square root of the corpus callosum area against the cubic root of brain mass derived from other cetaceans (blue dots; exponent of 0.946 ± 0.049 r^2^ = 0.85; p < 0.0001; Manger et al. 2010), the Guiana dolphin (*g) falls slightly below the 95% prediction interval for the group with an exponent of 0.956 ± 0.049; r^2^ = 0.85; p < 0.0001, having smaller than the expected cross-sectional area of the corpus callosum for its brain mass, **b** when regressing the gyrification index (GI) against the brain mass calculated derived from other cetaceans, the Guiana dolphin and all other cetaceans lie above the GI regression described for carnivores, artiodactyls, and primates (black line and dots; Manger et al. 2012). However, the Guiana dolphin's GI also falls substantially below the data points of other cetaceans (blue line; 0.019 ± 0.018; r^2^ = 0.215; p < 0.0003; data from Manger, 2012), making it an outlier when compared to both cetaceans and other mammals, **c** when regressing the hippocampus area against the brain volume, the Guiana dolphin (*g) falls well within the 95% prediction interval established for all the other cetaceans (exponent of (0.553 ± 0.168; r^2^ = 0.55; p = 0.0131; re-plotted from Patzke et al. 2015). The relationship that describes the regression of the hippocampus against brain volume for all the other non-cetacean mammals (black line) is better characterized by an exponential curve (red dashed lines), **d **when regressing the amygdala volume against the brain volume, the guiana dolphin (*g) falls well within the 95% prediction interval described from the regression derived for cetaceans, with an exponent of 0.813 ± 0.042 (blue line; r^2^ = 0.99; p < 0.0001), falling below the regression derived for all the other non-cetacean mammals (black line; exponent of 0.713 ± 0.005; r ^2^ = 0.98; p < 0.0001; Patzke et al. 2015), **e** when regressing the cerebellum volume against the brain volume, the Guiana dolphin (*g) falls well within the 95% prediction interval derived from this regression for other cetaceans with an exponent of 1.003 ± 0.033 (r.^2^ = 0.981; p = 0.0001; data from Marino et al. 2000; Maseko et al. 2012; Schwerdtfeger et al. 1984). In comparison to other mammals (primates, megachiropterans, and insectivores), cetaceans—including the Guiana dolphin—have larger than expected cerebellar volumes according to brain mass (data from Stephan et al. 1981; Baron et al. 1996; Schwerdtfeger et al. 1984; Marino et al. 2000), **f** When regressing the ventricular volume against the brain volume, the Guiana dolphin (*g) falls within the range described for other mammals (megachiropterans, microchiropterans, and insectivores; data from Stephan et al. 1981; Baron et al. 1996)

The amygdala complex lies in the medial part of the temporal lobe ventral to the most caudal region of the putamen (Figs. 4f, g; 5b; 6f). A clear delineation of the amygdalar subdivisions from our MRI scans was not possible. The amygdala volume was 0.95 cm^3^, which accounts for approximately 0.14% of the total brain volume. These values fall well within the 95% prediction interval described from the regression derived for cetaceans, which has an exponent of 0.813 ± 0.042 (r^2^ = 0.99; p < 0.0001), falling below the regression derived for all the other non-cetacean mammals (exponent of 0.713 ± 0.005; r^2^ = 0.98; p < 0.0001; Fig. 7D; data from Patzke et al. 2015).

The hippocampus is located caudal to the amygdala in the medial part of the temporal lobe (Figs. 4h; 5b; 6e,f). The volume calculated for the hippocampus was 0.66 cm^3^, which corresponds to approximately 0.10% of the total brain volume. This falls well within the 95% prediction interval from the relationship between hippocampal volume and brain volume established for cetaceans (0.553 ± 0.168; r^2^ = 0.55; p = 0.0135; Fig. 7c; data from Pirlot and Nelson 1978; Stephan et al. 1981; Baron et al. 1996; Reep et al. 2007; Montie et al. 2008; Patzke et al. 2015). The fornix, a white matter bundle that connects the hippocampus with the hypothalamus and other subcortical structures (Figs. 4d, e; 5d; 6c), is rather thin in this species.

### Commissures

The two cerebral hemispheres are connected by commissural fibers, with the largest bundle of commissural fibers forming the corpus callosum (Figs. 3e; 4b–h; 5d–f; 6b–f). As with other cetaceans, the corpus callosum (CC) of the Guiana dolphin is notably thin, which is reflected in its small mid-sagittal cross-sectional area (CCA) of just 1.132 cm^2^. This places the Guiana dolphin among cetaceans with the smallest absolute CCAs recorded. To our knowledge, only a single individual from the common dolphin species (*Delphinus delphis*) has been reported with a smaller CCA (Fig. 7a; data from Tarpley and Ridgway 1994; Manger et al. 2010).

The CCA is very close to what would be predicted based on the regression derived from other cetaceans. It falls within the 95% prediction intervals (0.956 ± 0.049; r^2^ = 0.85; p < 0.0001; blue line; Fig. 7a; data from Tarpley and Ridgway 1994; Keogh and Ridgway 2008). However, all cetaceans, including the Guiana dolphin, fall below the 95% prediction interval of the regression derived for other mammals. This indicates that cetaceans have a corpus callosum that is smaller than what would be expected for other mammals of the same brain mass. This finding suggests that, despite the Guiana dolphin having one of the smallest recorded absolute sizes of the corpus callosum among cetaceans, its relative size is typical for species within the delphinids (Fig. 7a).

Cetaceans typically possess a thin anterior commissure, both in absolute and relative terms. This is attributed to the regression of the olfactory system (Oelschläger et al. 2008). Probably due to its thinness, we could not identify this structure in our images.

At the diencephalic level, a well-developed posterior commissure was reported, which seems to be a typical feature for dolphins (Oelschläger et al. 2008, 2010). It is positioned above the most dorsal part of the tegmentum of the mesencephalon, connecting its constituent nuclei (Figs. 4g; 5e, f; 6c, d). Usually dorsal to the posterior commissure, the habenular commissure constitutes the commissural complex found at the epithalamic level (Figs. 4f; 5e; 6c) (Oelschläger et al. 2008).

## Ventricular system

The ventricular system follows the general morphology of the brain, being short longitudinally but wide laterally. The telencephalic hemisphere rotation in cetaceans made the lateral ventricles more semicircular in shape, with a short anterior horn when compared to other mammals (Mcfarland 1969). In contrast, the inferior horn appears as a wide structure, reflecting the expansion of the temporal lobes. No occipital horn could be observed.

We also observed the choroid plexus, which is a network of highly vascularized tissue located within the ventricles that produces cerebrospinal fluid (Figs. 4h; 5b; 6a). The septum pellucidum appears as a sheet running from the corpus callosum down to the fornix, separating the lateral ventricles medially (Figs. 4c; 5f; 6e,f). The third ventricle, situated at the diencephalic level surrounding the adhesion interthalamica, is continuous with the cerebral aqueduct caudally (Figs. 4f,g; 5f; 6d–f). The cerebral aqueduct (Figs. 4h–j; 5f; 6d) appears as a narrow canal ventral to the tectum, connecting the third and fourth ventricles, which in turn expand into lateral (Foramen of Luschka) and medial (Foramen of Magendi) apertures into the subarachnoid space.

For the volumetric analysis of the ventricular system, we delineated the two lateral, third, and fourth ventricles and the cerebral aqueduct. The calculated ventricular volume from our scans was 6.263 cm^3^, corresponding to 0.95% of the total brain volume. A strong correlation has been observed between ventricular volume and brain mass in groups such as megachiropterans, microchiropterans, and insectivores (Maseko et al. 2011; data from Stephan et al. 1981; Baron et al. 1996). Using these findings as a baseline for comparisons, our data indicate that the Guiana dolphin falls within the range described for these groups, exhibiting neither a particularly large nor small ventricular system relative to its brain mass (Fig. 7f). For other cetaceans, the only data we found was from McFarland (1969), which reported volume measurements of 14.0 and 9.5 cm^3^ for the ventricular system of the bottlenose dolphin (*Tursiops truncatus),* depending on the methodology applied*.*

## Diencephalon

The diencephalon is located between the telencephalon and the brainstem. Due to the rostroventral rotation of the hemispheres, the diencephalon is also ventrally rotated in its position. The voluminous thalamus occupies the largest proportion of the diencephalon (Figs. 4d–i; 5b–f; 6b–e), whereby the left and right thalamus are connected by a large interthalamic adhesion at their medial surfaces (Fig. 3e). As reported by Kruger (1959) for the bottlenose dolphin, we similarly observed a downward displacement of the caudal pole of the thalamus in the Guiana dolphin, attributed to the distinctive flexure of the mesencephalon. The volume calculated for the thalamus was 15.04 cm^3^, corresponding to 2.28% of the total brain volume, which is larger in comparison with the previous report of 9.453 cm3 for the *Tursios truncatus’* thalamus (Kruger 1959). The maximum transverse diameter measured here for both thalami was approximately 6.3 cm, comparable to the 7 cm reported for the bottlenose dolphin (Morgane and Jacobs 1972; Morgane et al. 1980).

Thalamic nuclei, including the pulvinar, medial geniculate body, and lateral geniculate body were evident from the MRI scans (Figs. 4h,i; 6b). The medial geniculate body is larger in comparison to the lateral geniculate body, emphasizing the importance of the auditory system in these animals. However, the lateral geniculate body, while smaller, is also well-developed and can be easily identified due to its distinct striped pattern visible in the MRI scans.

The hypothalamus is located ventrocaudal to the thalamus, surrounding the ventral portion of the third ventricle. Its rostral part lies above the optic chiasm, while the caudal part includes the mammillary bodies. The mammillary body was notably smaller compared to that of terrestrial mammals such as artiodactyls, their closest relatives (Figs. 4g; 5f; 6g; Cozzi et al. 2017). Due to the fragile location of the pituitary gland within the sphenoid bone, it was not feasible to preserve the integrity of the gland while removing the brain from the skull. However, the part of the structure that connects the hypothalamus to the pituitary gland—the infundibulum—could be identified at the base of the brain, close to the optic chiasm (Figs. 3d,e; 4g; 5f).

The dorsal part of the diencephalon comprises the small epithalamus, formed by the habenular complex, the pineal region, the pretectal area, and the paraventricular nucleus. The habenulae nuclei and the habenula commissure constitute the habenular complex, recognized as a well-developed structure in cetaceans (Oelschläger et al. 2008; Kruger 1959). The habenular complex, alongside the pineal gland, is situated near the midline of the brain, bordering the third ventricle (Figs. 4f; 5e; 6c). Despite previous reports of the absence of the pineal gland in certain dolphin species (Oelschläger et al. 2008; Holzmann 1991; Panin et al. 2012), we were able to observe this structure in all three planes of our acquisition (Figs. 4f, 5f, 6c; supplementary material). From the most mid-sagittal plane, the pineal gland transitions from a thin and elongated to a more robust and ovoid structure towards the most caudal part, rostral to the posterior commissure. In the horizontal and coronal planes, the form ranges from an ovoid shape to a more rectangular form towards the most ventral and caudal parts, respectively.

## Brainstem

The brainstem—comprised of mesencephalon, pons, and medulla oblongata—is continuous caudally with the diencephalon through the mesencephalic flexure. Of the brain’s flexures, the mesencephalic is the most pronounced and it allows the brainstem to align to the rostro-caudal axis. The total structure volume was 25.01 cm^3^, corresponding to 3.80% of the total brain volume. Individual brainstem structures, such as the superior and inferior colliculi, were also delineated from our scans (Table 1).

The mesencephalon is anatomically divided into the tectum, tegmentum, and crus cerebri. The tectum includes the superior and inferior colliculi composing the quadrigeminal plate. These ovoid structures are readily observed in all three planes of the acquisition (Figs. 4h–k; 5d, e; 6c, d). In cetaceans, the two inferior colliculi are more developed than the superior colliculi, corroborating the importance of auditory sensation in the group. The two sides of the inferior colliculi are connected in its most dorsal part by the commissure of the inferior colliculi (Figs. 6c; 4j). For the Guiana dolphin, the volume of the inferior colliculi was 2.68 cm^3^, approximately five times larger than the superior colliculi with a volume of 0.512 cm^3^ (Table 1). The volumes for each side of the inferior colliculi (left side: 1.352 cm^3^; right side: 1.211 cm^3^) closely align with those reported for the bottlenose dolphin’s inferior colliculi, measuring 1.775 cm^3^ and 1.345 cm^3^ and 1.451 cm^3^ (Orekhova et al. 2022**)**

In the tegmentum, one of the most evident structures is the large elliptic body (Figs. 4g,h; 5e,f; 6e,f). The structure appears to be unique to cetaceans and elephants, which are distantly related groups. Although its function remains unclear, it has been suggested to be involved in the control of facial movements (Morgane and Jacobs 1972), such as those related to the differentiated nasal structures in these animals – i.e., the blowhole in cetaceans and the trunk in elephants. The tegmentum also includes the brachium of the inferior colliculus, a thick band of auditory fibers that extends rostrally from the inferior colliculi to the medial geniculate body (Figs. 4i; 5c; 6d), as well as the well-developed reticular formation (Figs. 4j,k; 5e; 6f), and the periaqueductal gray (Figs. 4h–j; 5f; 6d).

Situated at the base of the mesencephalon, the crus cerebri appears as a semilunar-shaped, robust fiber bundle that forms part of the cerebral peduncle (Figs. 3d; 4h,i; 6f). Lying dorsal to this structure is the substantia nigra, which plays a crucial role in modulating motor function (Figs. 4i; 5f; 6e).

Caudal to the midbrain is the pons, which appears as a very massive and protruding structure (Figs. 3 d,e; 4h–l; 5d,f; 6g,h). The trapezoid body and the superior olivary nuclei form the superior olivary complex (Figs. 3d, e; 4l, m; 5d, e; 6g,h) are located in the caudal region of the pons. The lateral lemniscus (Figs. 4j,k; 5d; 6e–g), a thick fiber bundle that runs from the superior olivary complex and merges into the inferior colliculi, was identified. Additionally, the medial lemniscus, a structure related to exteroceptive sensitivity, is thin in comparison to the well-developed lateral lemniscus (Figs. 4 j, k; 5f; 6e–g).

The most caudal division of the brainstem is the medulla oblongata, which is continuous with the spinal cord. The inferior olivary complex is well-developed and bulges at the ventral surface of the medulla oblongata (Figs. 3d,e; 4n; 5d,e; 6h). Other components related to auditory processing–such as a robust vestibulocochlear nerve–were also observed (Figs. 3b,d; 4l; 5c; 6h).

## Cerebellum

The cerebellum of the Guiana dolphin conforms to the general pattern of organization that is found across mammalian species. It is notable for its gross morphological complexity relative to the cerebrum as it consists of extensive foliation partitioned into 3 lobes–the anterior, posterior, and flocculonodular lobe–that are further subdivided into 10 lobules by deep fissures (Fig. 5g). Structurally, the cerebellum consists of two large, laterally-projecting hemispheres that are connected by a narrow midline structure called the vermis (Fig. 3c,d). Due to the complexity of the cerebellar foliations, interpretation of the cetacean cerebellar fissures is contingent upon embryological data; thus, the cerebellar delineations presented herein are principally based upon the embryologically-guided delineations across extant cetacean species presented by Larsell (1970).

From MRI scans, we observed the cerebellar hemispheres, the vermis, as well as clusters of cerebellar nuclei (Figs. 4h–n; 5b–f; 6a–h). All major fissures separating the ten lobules of the cerebellum were also observed and delineated (Figs. 4k–n; 5e,g). Additionally, we identified the superior, middle, and inferior peduncles that connect the cerebellum to different parts of the brainstem (Figs. 4k–n; 5c–f; 6e,g).

Consistent with the general pattern of the mammalian cerebellum, the anterior and posterior lobes are separated by the primary fissure, whereas the posterior and flocculonodular lobes are separated by the secondary fissure (Figs. 4k–m; 5g). The anterior lobe is comprised of lobules I, II, III, IV, and V, whereas the posterior lobe consists of lobules VI, VII, VIII, and IX. Lobule X makes up the flocculonodular lobe. All ten lobules have a vermal (midline) and hemispheric (lateral) portion with the exception of lobules I–III, whose hemispheric projections are diminutive or absent (Fig. 5g). As is typical of cetaceans, the anterior lobe and flocculonodular lobe are small in comparison to the very large posterior lobe, which is dominated in this species by the well-developed paraflocculus (Figs. 3e; 4n; 5g; 6f).

In the median sagittal section, the anterior lobe comprises approximately one third of Guiana dolphin’s cerebellar vermis (Figs. 3e; 5g). Consistent with the findings of Larsell (1970), lobules I, II, and III are relatively large in the vermal (midline) cerebellum with respect to that which is typically found across mammalian species; however, the hemispheric extensions of these lobules are trivial. Lobule I projects into the anterior part of the fourth ventricle and is separated from lobule II by a deep precentral fissure (Fig. 5g). There are two surface lamellae present on lobule III. It is distinguished caudally from lobule II by the precentral fissure and from lobule IV rostrally by the preculminate fissure. Lobule IV is subdivided into two large vermal subdivisions, lobules IVA and IVB, that extend laterally to form a small, triangular hemispheric lobule IV. Lobule IV is separated from lobule V posteriorly by a deep intraculminate fissure. Lobule V consists of three superficial surface lamellae that are flanked posteriorly by additional small lamellae that project deep into the anterior wall of the primary fissure. Due to the diminutive size of the hemispheres of the anterior lobe, cetaceans have the smallest relative anterior lobe size of any group of mammals. Our observations of the Guiana dolphin cerebellar lobes are consistent with this finding.

Lobule VI of the posterior lobe is defined anteriorly by the presence of the deep primary fissure and posteriorly by the superior posterior fissure (Fig. 5g). Lobule VII, which is situated between the superior posterior and the prepyramidal fissures, has relatively small hemispheric extensions compared to the much more robust lateral extensions of the paraflocculus. It has been previously posited that the reduction of exposed surfaces of the of hemispheric lobule VII is due to a partial rostrocaudal compression of the cerebellum in cetaceans (Larsell 1970). In the midsagittal section, the two subdivisions of the vermis of lobule VIII, lobules VIIIA and VIIIB, extend dorsocaudally from the same medullary ray (Fig. 5g). Lobule VIII is defined anteriorly by the prepyramidal fissure and posteriorly by the secondary fissure whilst being split into its two aforementioned subdivisions by the presence of the intrapyramidal (or intrabiventer) fissure, whereas lobule IX is situated in between the prepyramidal and secondary fissures. In cetaceans, the hemisphere of lobule VIIIB corresponds to the dorsal paraflocculus whereas the hemisphere of lobule IX corresponds to the ventral paraflocculus (Larsell 1970). The paraflocculus, which is known to play a role in auditory function (Mennink et al. 2020), is especially enlarged in the Guiana dolphin. Moreover, lobules VIII and IX are characterized by a high degree of asymmetry. This is consistent with findings from other species of Delphinidae (Hanson et al. 2013).

The flocculonodular lobe, comprising a single lobule X, is delineated from the posterior lobe by the presence of the posterolateral fissure (Fig. 5g). Like in all cetaceans, lobule X is relatively small in comparison to the lobules of the posterior lobe.

The Guiana dolphin's cerebellar volume measured 113.82 cm^3^, accounting for 17.27% of its total brain mass. This lies within the range of 15–19% previously reported for marine dolphins (Pilleri and Gihr 1970).

Regarding the relationship between cerebellum volume and brain mass, the Guiana dolphin falls well within the 95% prediction interval derived from the regression for odontocetes with an exponent of 1.003 ± 0.033.1.003 ± 0.033 (r^2^ = 0.961; p < 0.0001; data from Marino et al. 2000; Maseko et al. 2012; Schwerdtfeger et al. 1984). This indicates that the cerebellar volume of the Guiana dolphin is what would be predicted for a cetacean of its brain mass. However, in comparison to other mammals’ regression (primates, megachiropterans, and insectivores), delphinids, including the Guiana dolphin, present an apparent shift, thus exhibiting larger than expected cerebellar volume to brain mass ratios (Fig. 7e; data from Stephan et al. 1981; Baron et al. 1996; Schwerdtfeger et al. 1984; Marino et al. 2000). It is worth noting that more recent research reports that some odontoceti and mysticeti species have smaller relative cerebellar volumes compared to delphinids (Ridgway et al. 2017; 2019).

## Discussion

The transition to a fully aquatic lifestyle led to unique morphological adaptations in cetacean anatomy, particularly in the brain. These adaptations are of significant importance from both evolutionary and comparative neuroanatomical perspectives. However, despite increasing interest from the scientific community, our understanding of cetacean brains remains incipient. This lack of comprehensive knowledge can be attributed to the difficulties associated with studying these free-ranging animals, as well as to the logistical challenges tied to collecting and analyzing cetacean brain samples. Moreover, the Order Cetacea exhibits an extensive variety in species, ecology, and geographical distribution (Fordyce 2018), making it a remarkably diverse clade. Consequently, the available neuroanatomical data for many species is limited, posing a significant challenge for conducting detailed comparative morphometric analysis.

We present here the first MRI-based description of the Guiana dolphin's brain, employing Ultra-High-Field Magnetic Resonance Imaging. Considering the scarcity of studies on the brains of different cetaceans, we hope that this work can serve as a template for future descriptions of cetacean brain morphology. Indeed, this brain was obtained in the context of the Brazilian Neurobiodiversity Network, which we recently established in part to remediate this lack of detailed descriptions of cetacean (and in particular Brazilian cetacean) brains. We thus hope to produce similar descriptions of other Brazilian cetacean species in the near future.

## Anatomical considerations–the Guiana dolphin as a delphinidae member

The Guiana dolphin belongs to the Delphinidae family and exhibits several similarities in terms of brain structure with its counterparts. Briefly, its brain is wider than it is long, and it is composed of a thin and highly folded cortical sheet of gray matter. The cerebral hemispheres are linked by a thin corpus callosum, and the cerebellum is large and situated dorsal to the caudal aspect of the short and robust brainstem. The striatum ensemble is well-developed and is located deep within the brain. The limbic structures, such as the hippocampus and amygdala, are relatively small, while the auditory structures are hypertrophied. As is typical of cetaceans, no evidence of olfactory structures was found in the Guiana dolphin brain.

Importantly, our study was one of the few full brain analyses in cetaceans to include a detailed characterization of the cerebellum, including delineations of all ten lobules. Given the typical focus on neocortical anatomy as well as the paucity of detailed comparative cerebellar data across species, we hope that this study sets the foundation for future studies to incorporate more detailed neuroanatomical considerations of the cerebellum. Considering the convergent evolution of many behaviors between cetaceans and humans, as well as the many avenues of evidence recently highlighting the role of the cerebellum in cognitive functions (Schmahmann 2019; Buckner 2013), we contend that the cetacean cerebellum presents ample opportunities for exploring the evolution of complex cognition in a comparative context.

Additionally, we identified neuroendocrine structures such as the pineal gland. It plays a crucial role in the synthesis and secretion of melatonin, and its shape may vary across cetacean species and other mammals. Morphological analysis, including MRI, has yielded inconsistent results regarding the presence of the pineal gland in cetaceans. While some studies have reported its presence (Montie et al. 2007; Wright et al. 2017), others have either failed to identify the structure (Marino 2001a, 2004b; Oelschläger et al. 2008, 2010) or have only noted its presence in a rudimentary form (Fuse 1936; Panin et al. 2012). The reasons for these variations remain unknown, but one hypothesis suggests that it may be related to sexual maturity and its role in the development of genital organs (Behrman 1990).

More recent studies posit that all cetaceans experience a loss of melatonin synthesis, regardless of the presence of the pineal gland (Lopes-Marques et al. 2019). Since melatonin plays a key role in regulating the sleep–wake cycle and other physiological processes related to circadian rhythms, the absence of melatonin prevents the pineal gland from mediating the melatonin-mediated circadian rhythms in cetaceans. This finding is consistent with an adaptation for unihemispheric sleep, which is exclusive to cetaceans (Emerling et al. 2021; Huelsmann et al. 2019; Valente et al. 2021). Nevertheless, it is important to note that, even when present, the pineal gland may be a vestigial structure (Valente et al. 2021), meaning that it either has no current function, its function is suboptimal or compromised, or it serves a different function than it did in the past (Werth 2014).

In contrast, the hypophysis (i.e., the pituitary gland) is a structure present in all living vertebrates (Harris 1950). In aquatic mammals, it has been suggested that this structure plays a key role in regulating body temperature (Cowan et al. 2008). This is of great importance since body heat is lost more rapidly while surrounded by water than by the atmosphere (Manger 2006). Previous studies of this structure in cetaceans have described it as large in absolute terms but small in relation to body size (Arvy 1971). Here, due to the fragility and location of the gland (Laios 2017), we had difficulties preserving it while removing the brain from the skull, and consequently, we could not observe it in the MRI scans.

Our analysis also revealed that the Guiana dolphin has one of the lowest corpus callosum absolute sizes of all cetaceans studied so far. The relative size, however, fits the observed scaling found in previous data for the group (Tarpley and Ridgway 1994; Manger et al. 2010). If we consider the role of the corpus callosum as the largest brain commissure connecting the cerebral hemispheres, the relatively small size of this structure in cetaceans (CCA: BM) supports the hypothesis of brain lateralization. This concept is further corroborated by the unihemispheric slow-wave sleep observed in cetaceans (Mukhametov 1985, 1987; Oleksenko et al. 1992; Lyamin et al. 2002).

Importantly, other factors beyond lateralization may also contribute to this pattern. Manger et al. (2010) proposed a hypothesis that the CCA: BM could be a result of smaller cerebral cortex sizes relative to the brain mass. Thus, additional studies are needed to clarify this issue and to elucidate what factors are involved in the small corpus callosum size in cetaceans, as exemplified by the specimen presented here.

In terms of cortical folding, cetaceans are known by their high gyrification indices (Hofman 1985; Manger et al. 2012). The Guiana dolphin exhibits a highly convoluted brain as seen in other Odontoceti species, with gyri concentrically organized around the Sylvian cleft. However, the gyrification index calculated for the Guiana dolphin is lower than the average of 5.43 reported for cetaceans (Manger et al. 2012), which may suggest a reduced cortical complexity in comparison to other odontocete species. Note, however, that the inclusion of the diencephalon in our segmentation of the Guiana dolphin brain increases the total and exposed areas used in this computation by the same amount, thus lowering the calculated GI value. Therefore, it is possible that the discrepancy observed was caused by methodological differences and not by a fundamental biological aspect.

We argue, however, that the segmentation used herein allows for a better comparative analysis between cetaceans, humans, and some non-human primates since it accounts for the intrinsic properties of the brain tissue represented in the MRI. By not including the diencephalon, the manual segmentation is severely limited by the contrast of the image and is more likely to present segmentation mistakes. The inclusion of the diencephalon permits the traced outline to largely coincide with a high-contrast transition, resulting in a less ambiguous segmentation. Indeed, with the likely future progress in automatic segmentation, this may become a better standard for comparison in a broader range of species as well.

Irrespective of the applied methodology, large variations in cortical gyrification across cetaceans have been previously observed when comparing mysticetes to odontocetes. For example, the bowhead whale *(Balaena mysticetus*) exhibits a low GI (2.32) compared to other cetaceans, yet its GI is comparable to artiodactyls, its sister group (Raghanti et al. 2019). However, the GI of the Guiana dolphin does not align with that of either cetaceans or artiodactyls, making it an outlier in terms of both absolute GI and the relationship between GI and brain mass.

To elucidate this question, it becomes essential to make use of more advanced imaging methods, notably MRI, to revisit, update, and broaden the available data on brain morphometric parameters, such as thickness and surface area for cetaceans. This would better explain how each morphological feature contributes to the gyrification process. Furthermore, exploring how gyrification occurs in different brain regions in cetaceans could be particularly significant in understanding their cortical complexity, which may also provide functional insights.

In the context of mammalian cortical development, previous investigations into the biomechanical properties of cortical gyrification suggest that it is specified almost fully by the cortical surface area and thickness of the cerebral cortex (Mota and Herculano-Houzel 2015), in accordance with a statistical physics-inspired model. In the model, cortical gyrification arises from the interplay between axonal mechanical tension, the hydrostatic pressure of the cerebral-spinal fluid, and the self-avoiding nature of cortical gray and white matter surfaces. Consequently, one should expect the degree of gyrification to increase with hydrostatic pressure.

Empirically, the cortices of all land mammals investigated follow a precise scaling law that is very close to what is predicted by the model, if one disregards the small variations in their ambient atmospheric pressure. The small number of cetaceans analyzed in the literature, however, seem to have a systematically higher degree of gyrification than that found in land mammals, even after considering their typically small cortical thickness and large cortical area. We thus postulate that this discrepancy is due to the increased hydrostatic pressures to which deep-diving marine cetaceans (which comprised all species then analyzed) are subjected to in their physical environment. To fully test this hypothesis, we need a broad set of both deep-diving and shallow-diving cetacean species; the present description of the Guiana dolphin is a first step along this research program, which we hope to extend to its sister species, and other river- and estuary-dwelling species. Ultimately, we hope to determine how environmental factors drive gyrification patterns across mammalian species throughout evolution.

## *Sotalia* evolution: a sister taxa relationship

The Guiana dolphin belongs to the genus *Sotalia*, which is part of the highly diverse family Delphinidae. The taxonomy of Guiana dolphins was controversial over history. Five species had been described: three riverine and two coastal species. Subsequent analysis reduced this number to two species: the marine *Sotalia guianensis* and the riverine *Sotalia fluviatilis* and later, they were grouped into a single species with riverine and marine ecotypes. Recent mitochondrial and nuclear DNA analyses associated with morphological studies separated the genus again into two different species: the marine *S. guianensis* and the riverine *S. fluviatilis* (Monteiro-Filho et al. 2002; Cunha et al. 2005; Caballero et al. 2007). Mitogenomic phylogenetic analysis dated their divergence at 2.3 million years ago (Cunha et al. 2011).

Although the marine and riverine ecotypes of *Sotalia* dolphins share many morphological similarities, their distinct evolutionary histories have resulted in some noticeable differences. They differ in skull shape and body size, and previous studies have also described variations in their development and reproductive aspects (da Silva and Best 1996; Flores et al. 2018).

Our understanding of the evolutionary relationships across cetacean species is limited in several respects. However, the findings presented here could serve as a foundation for future studies exploring which neuromorphological features are conserved or divergent between these two closely related species. Considering their distinct ecosystems, such analyses should provide valuable insights into how riverine or marine environments may influence cetacean brain anatomy.

## Methodological considerations

It is important to consider that the analyses presented here are limited by the inclusion of a single individual. Our limited access to a single Guiana dolphin brain sample highlights the rarity of such specimens as well as the challenges presented by collecting brain data from wild aquatic mammals. Nevertheless, the brain analyzed was obtained from a fresh carcass with no indications of abnormalities or pathology, thus presenting a rare opportunity to characterize the previously unknown neuroanatomy of the Guiana dolphin.

In terms of technical concerns, anatomical and volumetric studies can be susceptible to measurement errors arising from postmortem factors that occur between sample collection and improper image processing and acquisition. To overcome these challenges, MRI techniques represent an innovative and alternative way to study different aspects of the brain, ranging from gross morphology to pathologies, while preserving the integrity of both internal and external brain tissue. This is of particular significance for cetaceans, as their brains undergo a rostroventral rotation of the forebrain, resulting in the repositioning of structures. In this sense, the “virtopsy” approach has been demonstrated to be even more effective in preserving the structures of the cetacean brain as it enables the analysis of brains in advanced stages of decomposition (Tsui et al. 2020). Therefore, it offers a new avenue for studying the brain of cetaceans.

Previous research suggests that utilizing ultra-high-resolution imaging in cetacean brain studies could minimize measurement errors and enhance the accuracy of neuroanatomical data (Wright et al. 2017). In our analysis, we corroborated this suggestion by employing a 7T ultra-high field resonance machine, which represents an improvement upon the previously published method.

Our approach offers greater anatomical reliability of brain imaging, enabling us to observe a wider range of brain structures in remarkable detail with more accurate gray/white matter contrast. Moreover, imaging blurring has been substantially reduced, and we have been able to generate more reliable volumetric data. Complementarily, and very recently, Orekhova and colleagues (2023) presented the assessment of the auditory connectivity and auditory nuclei volumes in the bottlenose dolphin brain using ultra-high-field MRI, highlighting the diverse range of quantitative studies achievable with this methodology.

As an additional benefit, the neuroanatomical description of the healthy brain in this species establishes a baseline that can be used to investigate and characterize neuropathologies that may affect the central nervous system in cetaceans and to draw parallels with similar processes in humans. Specifically, morbilliviruses have been identified as potentially causing epidemics and mass mortality in the Guiana dolphin, highlighting the need for further research in this area (Cunha et al. 2021; Groch et al. 2018; 2020). Apart from morbilliviruses, other diseases could impact the brain, such as toxoplasmosis and neurobrucellosis, which have previously been reported in this species (Gonzales-Viera et al. 2013; Sánchez‐Sarmiento et al. 2019). It is common that in some studies the brain could not be examined due to advanced decomposition or the necessity of preserving the skull for cranial morphology studies or any other logistical issue. Consequently, many other potential brain pathologies remain unexplored in these animals. In cases where accessing the brain for histological and genetic examination is not feasible or practical, MRI studies offer an alternate and valuable approach to investigating neuropathologies in cetaceans. Through MRI studies, we can explore and investigate the potential causes of mortality in these animals, providing valuable insights that can enhance existing management and conservation strategies. The incorporation of ultra-high-field MRI in our investigation further increases the benefits of this technique since it offers the advantage of more precise visualization of pathologies that might be challenging or even impossible to detect using lower-field machines (Platt et al. 2021).

To estimate gray matter and white matter volumes of the cerebral cortex, we have applied a relatively new method we have developed (Ribeiro et al. 2013). Instead of multiplying the contour areas by slice separation for each slice and then adding them all together, as was done in previous studies (Elias and Schwartz 1969; Hofman 1985), our calculation allows for the varying slope in the lateral surface between adjacent brain slices. The differences in volume estimation between the two methods are not large and vanish as the slice separation goes to zero. However, for surface area estimation the traditional method introduces large systematic errors for any slice separation, which is absent in our method (see Avelino-de-Souza 2023, Appendix D for more details).

## Conclusion

Our research presents an unprecedented neuroanatomical dataset for the Guiana dolphin brain using a state-of-the-art, ultra-high-field machine—an approach that is still uncommon in cetacean brain studies. This study marks a significant advance in our understanding of the Guiana dolphin brain, and we hope it will serve as a template for the anatomical description of lesser-known cetacean species. Of particular interest, we hope to describe species that differ from the Guiana dolphin in their phylogenetic histories and physical environment: Starting with its river-dwelling sister species, the Tucuxi dolphin (*Sotalia fluviatilis*), and broadening the scope to include river-, estuary- and ocean-dwelling species. In this way, we can comprehensively test the hypothesis according to which ambient hydrostatic pressure directly and positively affects cortical gyrification.

More broadly, cetaceans are exceptional mammals, shaped by evolution in response to a unique physical, ecological, cognitive, and sensory environment. By shedding light on the brain structure of cetaceans and making comparisons with other mammals, we lay essential groundwork to understand how brain evolution in general is influenced by adaptation, physical constraints, and the vicissitudes of phylogenetic history.

## Supplementary Information

Below is the link to the electronic supplementary material.

Supplementary file1 (PDF 17496 KB)

## Data Availability

The original datasets produced and examined during this study can be obtained upon a reasonable request from the corresponding author. MRI images with no labeling are available as supplementary material.

## Abbreviations

a: Interthalamic adhesion
aq: Cerebral aqueduct
BW: Receiver bandwidth
CC: Corpus callosum
Cr: Cruciate sulcus
e: Elliptic body
Ec: Ectolateralis sulcus—Superior lateral fissure
En: Entolateral sulcus—Paralimbic cleft
Es: Ectosylvian fissure—Inferior lateral fissure
f: Fornix
FoV: Field of view
Inf: Infundibulum
intc: Intraculminate cerebellar fissure
IO: Inferior olive
ip: Intrapyramidal cerebellar fissure
is: Intercalate sulcus
La: Lateral sulcus
LH: Left hemisphere
mb: Mammillary body
pc: Posterior commissure
pcm: Preculminate cerebellar fissure
Pfl: Paraflocculus
pl: Posterolateral cerebellar fissure
pp: Prepyramidal/prebiventer cerebellar fissure
pri: Primary cerebellar fissure
RH: Right hemisphere
Sc: Spinal cord
scc: Sulcus of corpus callosum
sec: Secondary cerebellar fissure
sp: Superior posterior cerebellar fissure
SS: Suprasylvian sulcus—Intermediary lateral fissure
Ssp: Suprasplenial sulcus
SyS: Sylvian sulcus
T: Thalamus
Tb: Trapezoid body
Te: Tectum
TE: Echo Time
TR: Repetition Time
Ve: Vermis
1: 1st ventricle
2: 2nd ventricle
3: 3rd ventricle
4: 4th ventricle
I: Lobule I of the cerebellum
II: Lobule II of the cerebellum
III: Lobule III of the cerebellum
IVA and IVB: Subdivisions of lobule IV of the cerebellum
V: Lobule V of the cerebellum
VI: Lobule VI of the cerebellum
VII: Lobule VII of the cerebellum
