# Supplementary material for: Anatomical and volumetric description of the guiana dolphin (Sotalia guianensis) brain from an ultra-high-field magnetic resonance imaging
Source: Brain Struct Funct. 2024 Apr 25;229(8):1889–911. doi: 10.1007/s00429-024-02789-1 (PMC11485192; doi:10.1007/s00429-024-02789-1)

Title: Anatomical and Volumetric Description of the Guiana Dolphin (*Sotalia guianensis*) Brain from an Ultra-High-Field Magnetic Resonance Imaging

Journal: Special Issue: Towards multi-modal, multi-species brain atlases; Brain Structure and Function

Author(s): Kamilla Avelino-de-Souza, Heitor Mynssen, Khallil Chaim, Ashley Parks, Joana M. P. Ikeda, Haydée Andrade-Cunha, Bruno Mota, and Nina Patzke.

Corresponding Author:
K. Avelino-de-Souza
Instituto de Física, Universidade Federal do Rio de Janeiro, University City, 21941-909, Rio de Janeiro, Brazil.
E-Mail: kamilla_bio@ufrj.br
Corresponding Author:
N. Patzke
Faculty of Medicine, IMBB, HMU Health and Medical University, Olympischer Weg 1, 14471, Potsdam, Germany.
E-Mail: nina.patzke@health-and-medical-university.de

Supplementary information

S1: Calculating the lateral area between cortical contours of adjacent slices
S2: Surface area of a sphere
S3: Surface area of a truncated cone
S4: Coronal magnetic resonance imaging (MRI) scans of the Guiana dolphin brain with no labeling
S5: Axial magnetic resonance imaging (MRI) scans of the Guiana dolphin brain with no labeling
S6: Sagittal magnetic resonance imaging (MRI) scans of the Guiana dolphin brain with no labeling
S7: Representative slice of each of the 2D MRI acquisition (left) and the 3D MRI acquisition (right) prior to any contrast adjustments.
S8: Zoomed-in image of the pineal gland (star) in the Guiana dolphin (*S. guianensis*) in coronal, sagittal and axial planes, respectively.

Supplementary Information S1: Calculating the lateral area between cortical contours of adjacent slices

We have previously derived (Ribeiro et al. 2013) approximate formulas previously for the volume V_n and lateral surface area An of the section between two closed contours n and n+1 situated on parallel cortical slices separated by distance h. If P_n and P_{n+1} are their respective perimeters, enclosing areas S_n and S_{n+1} respectively, and assuming that (a) each section's contour is approximately congruent to that of its neighbors and that (b) the slice's lateral surface makes an approximately constant angle with the coronal plane. In this case, it can be shown that

$$A_n = \sqrt{\left[\frac{h(P_n + P_{n+1})}{2}\right]^2 + (S_n - S_{n+1})^2}$$
$$V_n = \frac{h}{3}(S_n + S_{n+1} + \sqrt{S_n S_{n+1}})$$

Previous works (Hofman 1985; Manger et al. 2012; Pillay and Manger 2007) did not account for the sloping of the lateral area, and simply assumed lateral surfaces descending vertically from each contour. This is equivalent to the alternate formulas

$$A_n^{Hof} = hP_n$$
$$V_n^{Hof} = hS_n$$

Note that the formulas coincide only if the adjacent contours are identical.

We will show below that if we apply the formulas above for two simple geometric shapes, namely the truncated cone and the sphere, in the limit where the slice separation h is very small, our formulas will converge to the correct expression for lateral areas, while the legacy formula will systematically yield substantially lower values.

Consider a set of circular contours centered at the z-axis of radius $\rho(z)$ at position z and separation $h = \Delta z$. Since we are assuming circular contours, then $S(z) = \pi\rho(z)^2$, $S(z+\Delta z) = \pi(\rho(z)+\Delta\rho)^2 \approx S(z) + 2\pi\rho(z)\Delta\rho$, $P(z) = 2\pi\rho(z)$ and $P(z+\Delta z) = 2\pi(\rho(z)+\Delta\rho)$. In the limit where Δz goes to zero, the formula for $A(z)$ can e written using the expressions above and retaining only terms linear in $\Delta\rho$ and Δz

$$A(z+\Delta z) - A(z) = 2\pi\rho(z)\sqrt{\Delta\rho^2 + \Delta z^2}$$

Since $\Delta\rho$ is a function of z, this implies in the limit:

$$\frac{dA}{dz} = 2\pi\rho(z)\sqrt{1+\left(\frac{d\rho}{dz}\right)^2}$$

Integrating over the entire surface we get:

$$A = 2\pi\int\rho(z)\sqrt{1+\left(\frac{d\rho}{dz}\right)^2}\,dz$$

Conversely, we can obtain the equivalent formula for the alternate lateral area by simply dropping the second term inside the square root:

$$A^{Hof} = 2\pi\int\rho(z)dz$$

Since $\sqrt{1+\left(\frac{d\rho}{dz}\right)^2} \geq 1$, then $A \geq A^{Hof}$ for all cases. Thus, the alternate formula always an underestimate of the correct area, except in the limiting case where the contours are identical (and thus $\frac{d\rho}{dz} = 0$). This proof can be generalized for a generic contour using vector calculus.

To compute these integrals explicitly and obtain the deficit, one needs to obtain for each case of interest the expression for $\rho(z)$, which we do below. As for the volumes, both formulas will converge to the correct value in the limit $\Delta z \to 0$, although the convergence will be faster with our formula.

Supplementary Information S2: Surface area of a sphere

For a sphere of Radius R, it is easier to compute the area of a hemisphere and double it. Let $z = 0$ correspond to the south pole and $z = R$ to the equator. Since two corresponding points will be at the same distance from the

center of the sphere, then clearly

$$(R-z)^2+\rho^2=R^2=(R-z-\Delta z)^2+(\rho+\Delta\rho)^2$$

Which, in the limit of $\Delta z \to 0$, reduces to

$$\frac{d\rho}{dz}=\frac{-R-z}{\rho}$$

And integrates to

$$\rho=\sqrt{2Rz-z^2}$$

Combining it all we obtain the correct area for the sphere

$$A_{sphere}=4\pi R\int_0^R dz=4\pi R^2$$

However, for the alternative area calculation we obtain

$$A_{sphere}^{Hof}=4\pi R^2\int_0^1\sqrt{2u-u^2}du=4\pi R^2\frac{\pi}{4}\approx 0.79A_{sphere}$$

Which is of course substantially lower than the correct value.

Supplementary Information S3: Surface area of a truncated cone

Let be r and R be the radii of the cone sections at heights $z=0$ and $z=H$ respectively. Then

$$\rho=r+(R-r)\frac{z}{H}$$
$$\frac{d\rho}{dz}=\frac{-R-z}{\rho}$$

Integrating, we obtain the correct value using our formula

$$A_{T.cone}=H(R+r)\sqrt{1+\left(\frac{R-r}{H}\right)^2}$$

While systematically underestimating the area for the alternate formula, except in the case where $r=R$, where both formulas coincide

$$A_{sphere}^{Hof}=H(R+r)$$

Supplementary Figure S4: Coronal magnetic resonance imaging (MRI) scans of the Guiana dolphin brain with no labeling

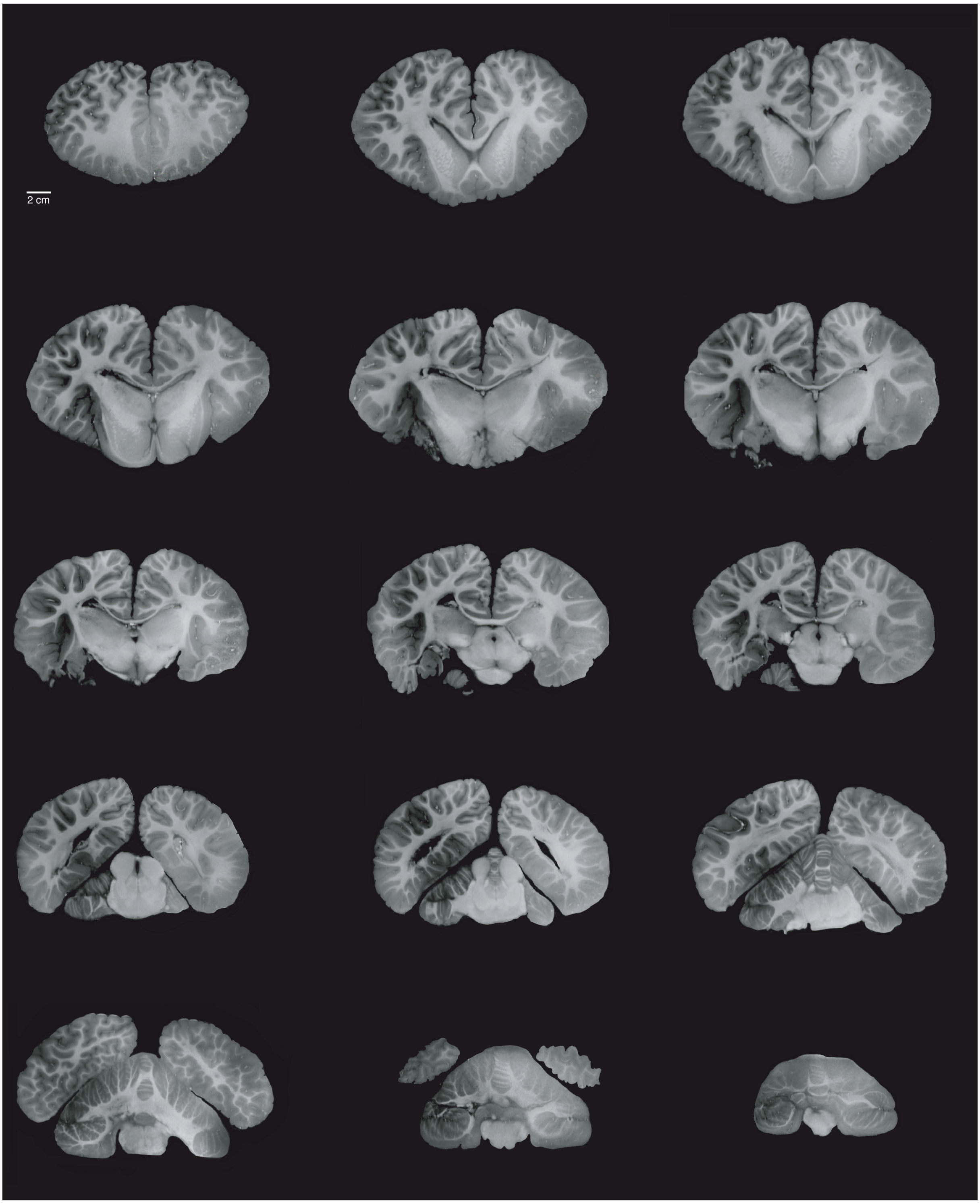

Supplementary Figure S5: Axial magnetic resonance imaging (MRI) scans of the Guiana dolphin brain with no labeling

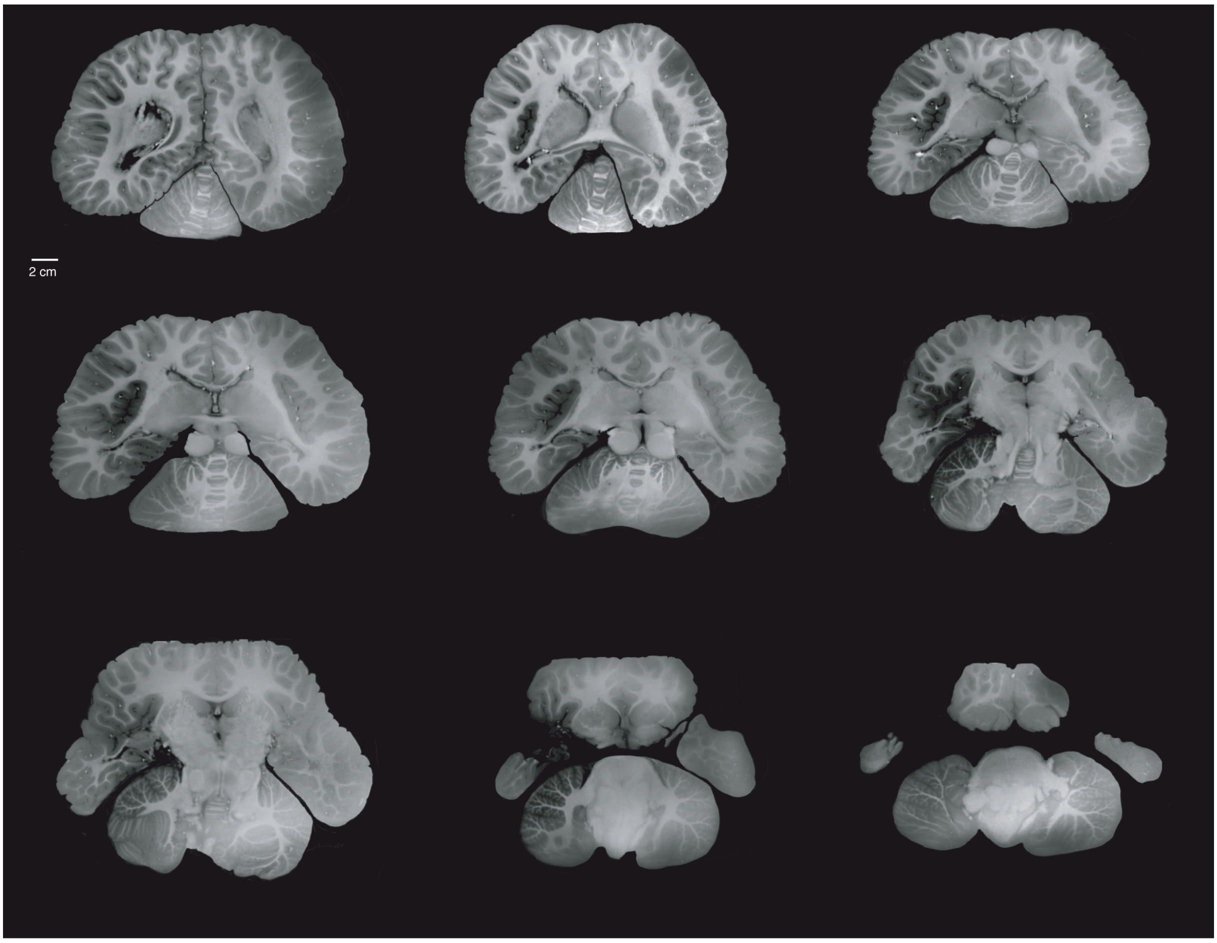

Supplementary Figure S6: Sagittal magnetic resonance imaging (MRI) scans of the Guiana dolphin brain with no labeling

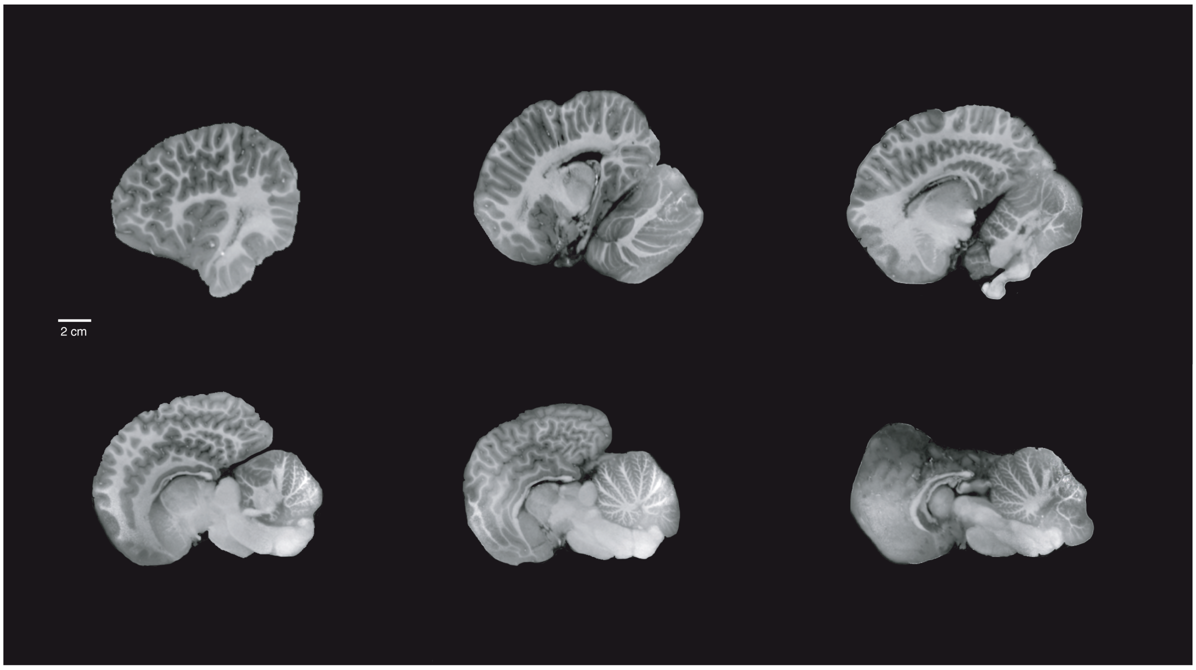

Supplementary Figure S7: Representative slice of each of the 2D MRI acquisition (left) and the 3D MRI acquisition (right) prior to any contrast adjustments.

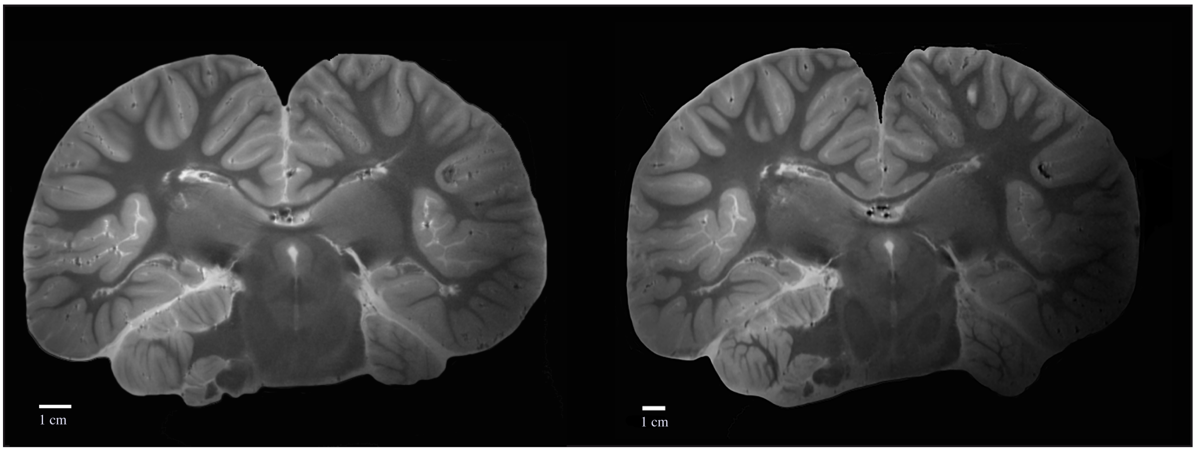

Supplementary Figure S8: Zoomed-in image of the pineal gland (star) in the Guiana dolphin (*S. guianensis*) in coronal, sagittal and axial planes, respectively.

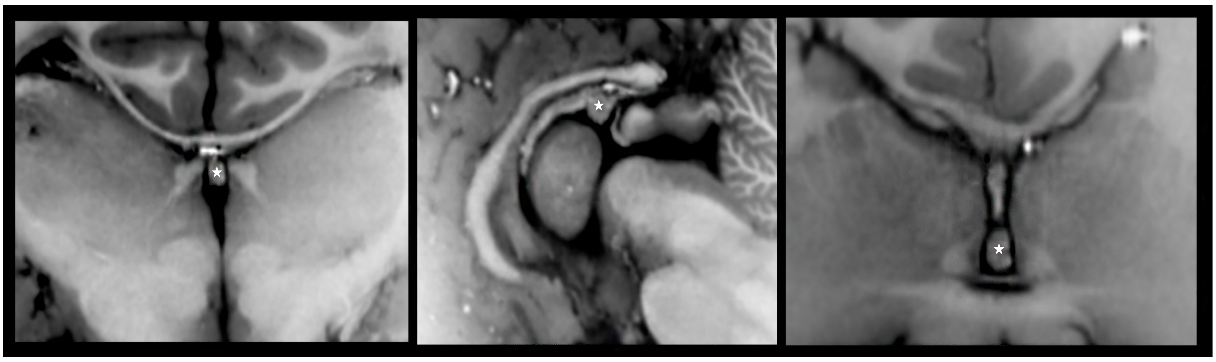

Supplement: Supplementary file 1 — Supplementary file1 (PDF 17496 KB) [file 429_2024_2789_MOESM1_ESM.pdf]
